# Microenvironment‐Guided Evolution of ssDNA‐SWCNT Probes for Selective Recognition of Aggressive Prostate Cancer Phenotypes

**DOI:** 10.1002/advs.202518582

**Published:** 2026-01-21

**Authors:** Dakyeon Lee, Seok‐Hyeon Lee, Yunseo Jung, Jeongho Lee, Min‐Seo Choi, SaeOck Oh, Sungjee Kim, Byoung Soo Kim, Sanghwa Jeong

**Affiliations:** ^1^ School of Biomedical Convergence Engineering Pusan National University Yangsan Republic of Korea; ^2^ Department of Chemistry Pohang University of Science and Technology Pohang Republic of Korea; ^3^ Department of Anatomy School of Medicine Pusan National University Yangsan Republic of Korea; ^4^ Research Institute for Convergence of Biomedical Science and Technology Pusan National University Yangsan Hospital Yangsan Republic of Korea

**Keywords:** 3D bioprinting, carbon nanotube, DNA, prostate cancer, SELEX

## Abstract

Despite advances in prostate cancer detection, distinguishing indolent from aggressive phenotypes remains challenging. We report a microenvironment‐guided strategy for evolving phenotype‐specific molecular probes using single‐stranded DNA‐functionalized single‐walled carbon nanotubes (ssDNA‐SWCNTs). Our approach employs 3D tumor models that recapitulate complex cancer microenvironments, enabling identification of ssDNA sequences with differential binding properties. We developed two distinct probes for prostate cancer cells: PC3D2, which preferentially binds hypoxia‐adapted stem‐like cells associated with treatment resistance, and PC2D2, which shows enhanced binding to mesenchymal‐like cells. These probes exhibit characteristic second near‐infrared (NIR‐II, 1000‐1700 nm) fluorescence, enabling non‐invasive detection of aggressive phenotypes in heterogeneous tumors using NIR‐II optical imaging. We demonstrate their utility for selective drug delivery to prostate cancer spheroids, resulting in enhanced therapeutic efficacy. This platform represents a significant advancement in precision diagnostics and theranostics, potentially transforming prostate cancer management through phenotype‐specific targeting. The methodology offers a generalizable approach for developing nanoprobes that recognize clinically relevant cancer phenotypes based on their unique microenvironmental signatures rather than individual biomarkers.

## Graphical abstract

Random ssDNA–SWCNT(single strand DNA‐single walled carbon nanotube) library is functionally screened and evolved using high‐throughput 3D prostate tumor spheroids via iterative positive and negative selection, enabling the identification of NIR‐II fluorescent ssDNA‐SWCNT nanoprobe specific for prostate cancer.

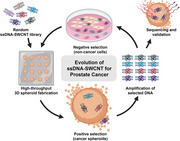

## 1 Introduction

Prostate adenocarcinoma is one of the most common malignancies in men worldwide and remains a leading cause of cancer‐related mortality despite the advances in diagnosis and treatment [1, 2]. Although early detection improves clinical outcomes, current screening and diagnostic methods show limited specificity, particularly in differentiating malignant tumors from benign prostatic conditions such as hyperplasia and prostatitis [3]. Prostate‐specific antigen (PSA) is the most widely used biomarker, expressed in normal and malignant prostate tissues and secreted into the seminal fluid [4], limiting its cancer specificity and making accurate risk stratification difficult.

Various molecular targeting strategies have been explored to overcome these limitations, including monoclonal antibodies [5], aptamers [6], nanobodies [7], and affibodies [8]. Antibodies offer high specificity and affinity to target proteins [3, 5], but they suffer from high production costs, batch‐to‐batch variability, degradation, and immunogenicity, which hinder their clinical application. As an alternative modality, aptamers—short single‐stranded DNA or RNA oligonucleotides that fold into specific tertiary structures—enable high‐affinity, high‐specificity binding to a wide range of targets, including proteins, small molecules, and whole cells [6, 9, 10, 11], low immunogenicity, chemical stability, scalable synthesis, and cost‐effectiveness. Aptamers can be generated through the systematic evolution of ligands by exponential enrichment (SELEX) process [12], an iterative in vitro selection process. In particular, Cell‐SELEX allows the selection against living cells without prior biomarker knowledge [13]. This approach has been applied successfully to generate prostate cancer‐specific aptamers, facilitating selective targeting, diagnostic imaging, and therapeutic delivery [14].

Despite the success of cell‐SELEX in 2D culture systems, these models do not adequately recapitulate the spatial and biochemical complexities of the tumor microenvironment (TME) [15, 16, 17]. The TME is strongly influenced by dynamic interactions with the extracellular matrix (ECM), hypoxia, cell–cell, and cell–matrix in tumor progression and therapeutic resistance [18, 19]. 3D culture models, such as spheroids and organoids, mimic the native tissue conditions more accurately by preserving the architecture, mechanical forces, hypoxic gradients, and signaling [20]. These systems enable more physiologically relevant screening platforms. Recently, 3D cell‐SELEX has been developed to recapitulate invasive and metastatic phenotypes [21, 22, 23], improving the clinical relevance of selected aptamer probes.

In parallel with aptamers, single‐strand DNA (ssDNA)‐wrapped single‐walled carbon nanotubes (SWCNTs) have emerged as a versatile molecular recognition platform. In this system, ssDNA adsorbed onto the nanotube surface forms a structured corona phase capable of selective analyte binding—a mechanism known as corona phase molecular recognition (CoPhMoRe) [24]. CoPhMoRe allows the near‐infrared (NIR) photoluminescence of SWCNT as a real‐time optical probe for molecular recognition. SWCNTs offer exceptional biocompatibility, optoelectronic properties, and intrinsic fluorescence in the NIR‐II window (900–1700 nm), enabling deep tissue imaging with minimal background interference, which is ideal for in vivo biosensing applications [25, 26, 27]. Williams et al. developed a fluorescent SWCNT nanosensor for metastatic prostate cancer detection by modulating the optical bandgap using ssDNA conjugated to a monoclonal anti‐urokinase plasminogen activator antibody [28]. Sores et al. fabricated an electrochemical ssDNA‐SWCNT biosensor targeting PCA3, a prostate cancer‐specific long non‐coding RNA, using complementary ssDNA and a layer‐by‐layer immobilization approach [29]. Lerner et al. introduced a SWCNT field‐effect transistor to detect osteopontin, functionalized with a single‐chain variable fragment protein to enhance the binding affinity and sensitivity [30]. Beyond diagnostics, SWCNTs are crucial in cancer therapy, enabling targeted drug delivery, reducing systemic toxicity, and synergizing with photothermal and photodynamic therapies [31]. SWCNTs offer a multifaceted approach to hindering tumor progression by targeting both tumor cells and the surrounding TME.

**FIGURE 1:**
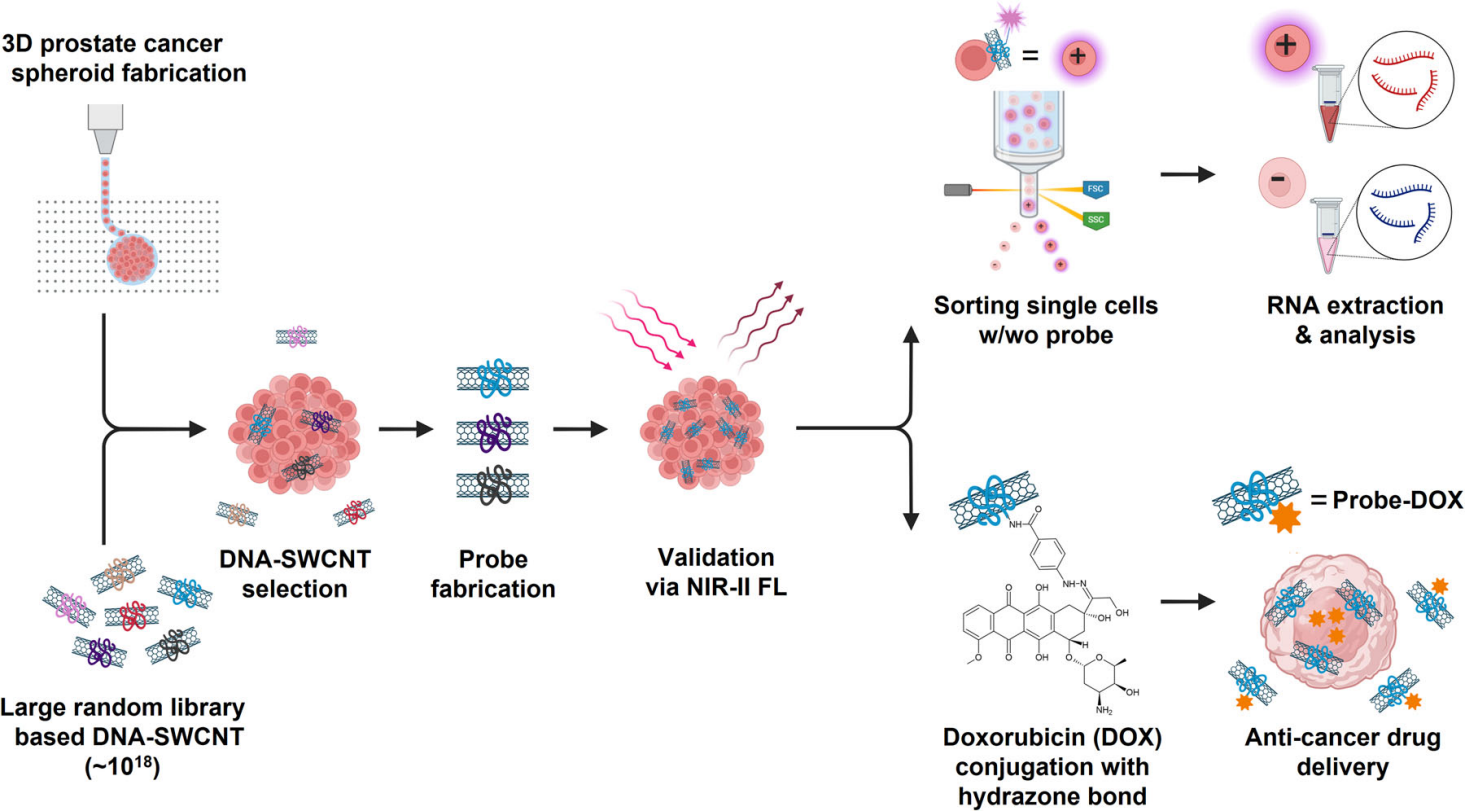
Workflow for selecting of ssDNA‐SWCNT library for prostate cancer probes using a 3D spheroid model. Screening of a large random ssDNA‐SWCNT library (∼10^1^ ^8^ sequences) was performed using a 3D bioprinted spheroid model of prostate cancer (LNCaP), enabling the identification of candidate probes with high binding affinity and specificity. After optimizing the targeting efficiency, the selected probes were validated by NIR‐II fluorescence (FL) imaging to assess selective accumulation and internalization within tumor spheroids. Probe–cell interactions were examined further by conducting single‐cell sorting (with or without probe binding), followed by RNA extraction and analysis to identify probe‐targeted subpopulations. In parallel, doxorubicin (DOX) was conjugated to the selected probes via pH‐sensitive hydrazone bonds to produce a theranostic probe–drug complex, which enabled targeted delivery and intracellular release of the anticancer agent. A schematic was produced using BioRender.

In this study, we present an integrated strategy to develop high‐affinity, prostate cancer cell‐specific molecular probes by combining ssDNA‐functionalized SWCNTs, high‐throughput systematic evolution of ligands by exponential enrichment after adsorption to carbon nanotubes (SELEC) screening (from ∼10^18^ unique sequences), and 3D bioprinted tumor models (Figure 1). Unlike conventional 2D screens, the proposed platform used 2D monolayers and 3D spheroids printed with decellularized ECM (dECM) bioinks to recapitulate the tumor microenvironment better. This model enables the selection of ssDNA‐SWCNT probes with enhanced binding specificity and cellular internalization. We assessed probe targeting and internalization using NIR‐II fluorescence imaging and fluorescence‐activated cell sorting (FACS)‐based single‐cell profiling to identify distinct subpopulations. Conjugation with chemotherapeutics has the potential for targeted drug delivery with reduced off‐target effects.

## 2 Results

### 2.1 Establishing a TME‐Mimetic Environment via Material Selection and In‐Bath Bioprinting

**FIGURE 2:**
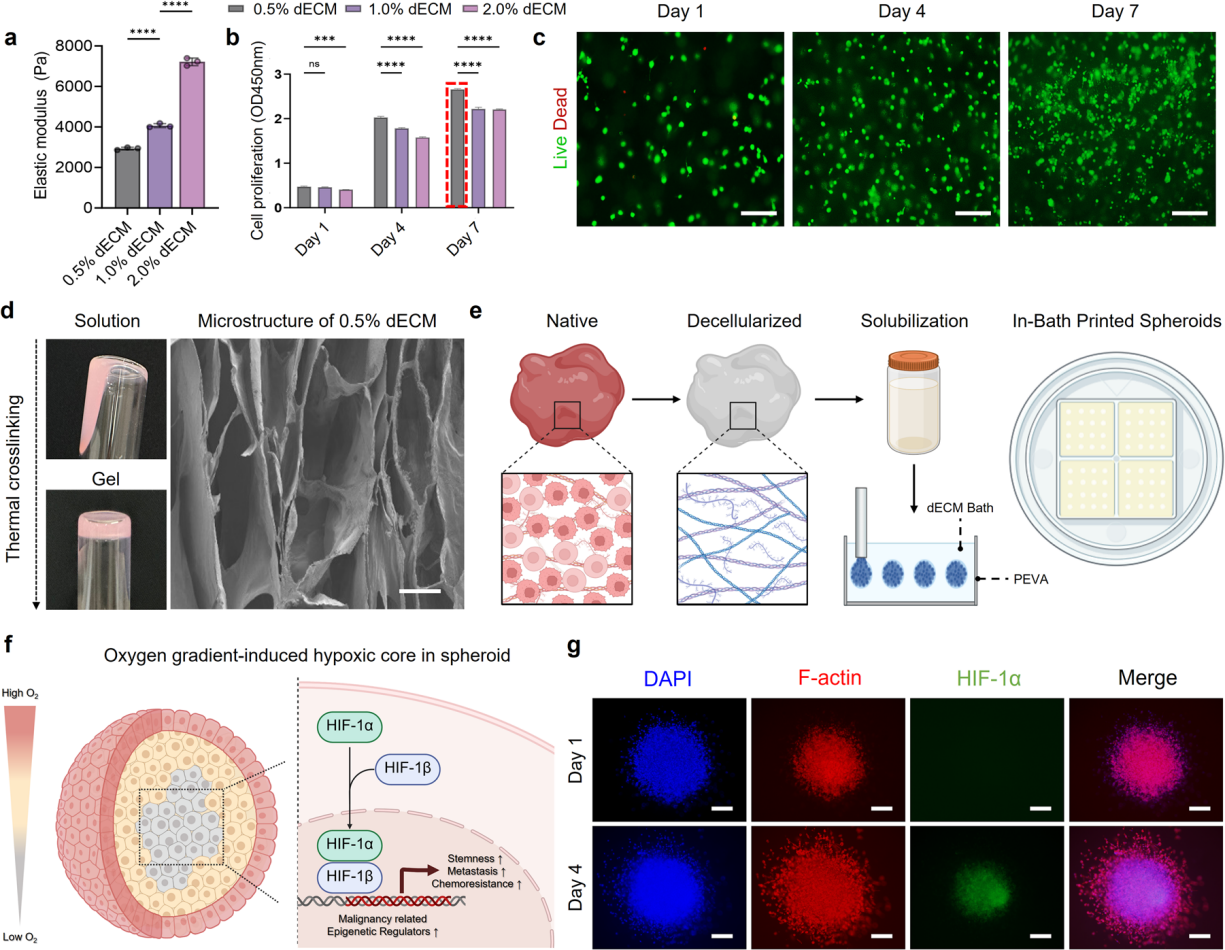
Structural, rheological, and mechanical analysis of 3D LNCaP spheroids printed with dECM bioink. (a) Elastic modulus of 0.5%, 1.0%, and 2.0% dECM. (b) LNCaP cell proliferation with varying dECM concentrations (0.5%, 1.0%, 2.0%) at days 1, 4, and 7. (c) Live/dead staining of encapsulated LNCaP cells in 0.5% dECM (green: live cells, red: dead cells). Scale bar, 100 µm.(d) Optical images (left) and SEM cross‐sectional images (right) of thermally cross‐linked 0.5% dECM at an accelerating voltage of 5 kV. Scale bar, 100 µm. (e) Schematic diagram of the fabrication process using decellularized ECM (dECM) for cell encapsulation and bath support. Tissue‐derived ECM was decellularized, solubilized, and combined with the cells to produce a bioink, which was then used for in‐bath bioprinting to produce structurally confined spheroid units. (f) Schematic illustration showing the formation of a hypoxic core within a spheroid induced by the oxygen gradient, and the hypoxia‐driven activation of malignant phenotypes. (g) Immunostaining images for comparison of HIF‐1α expression on days 1 and 4. Scale bar, 200 µm In a, b, e, and h, bar graphs represent mean values with error bars indicating standard deviation (s.d.). Statistical significance was determined by ordinary one‐way ANOVA using multiple comparison tests. (^*^ *p* <0.05; ^**^ *p* <0.01; ^***^ *p* <0.001; ^****^ *p* <0.0001). The schematic was produced using BioRender.

The TME of prostate cancer was mimicked by fabricating, 3D LNCaP spheroids using in‐bath bioprinting with dECM bioink. This method enables direct cell printing within a pre‐gel matrix that mimics the ECM and supports spheroid formation with high precision and reproducibility [32, 33]. The spheroid size was regulated by the printing pressure, and the spatial positioning was controlled via G‐code (Figure S1). dECM was selected as the bath material due to its collagen and glycosaminoglycan (GAG) content and low immunogenicity (Figure S2) [34, 35]. Bioink characterization was performed to optimize the printing parameters. The elastic modulus of dECM increased with concentration: 0.5% [2933.33±66.58 Pa], 1.0% [4066.33±99.12 Pa], and 2.0% [7219.34±186.43 Pa] (Figure 2a). Subsequently, LNCaP cells were encapsulated within each dECM bioink formulation to evaluate their proliferation dynamics. Quantitative analysis revealed stiffness‐dependent variations in cell proliferation, with a statistically significant increase observed in the 0.5% dECM bioink compared to the higher‐stiffness groups (Figure 2b). Previous studies have reported that LNCaP cells exhibit enhanced proliferative and functional responses under relatively soft, physiologically relevant microenvironments, accompanied by androgen activation and CD44 clustering [36, 37, 38, 39, 40]. Consistent with these findings, our system exhibited higher proliferation rates under the relatively soft dECM bioink. Furthermore, the elastic modulus of the 0.5% dECM bioink (2933.33 ± 66.58 Pa) was found to be similar to the stiffness range reported for normal prostate tissue [41, 42, 43]. While softer matrices may further promote proliferation, bioink formulations must also maintain sufficient mechanical integrity to ensure stable printability in the in‐bath bioprinting process, which requires a minimal stiffness threshold as demonstrated in our previous study [32, 44]. Collectively, considering the biophysical properties, cellular functionality, and printability requirements, the 0.5% (w/v) dECM bioink was selected as the optimal condition for subsequent experiments. Live/Dead staining was performed over a seven‐day culture period to assess the cytocompatibility of the selected bioink. The results confirmed minimal cell death, suggesting that the bioink provided a supportive and cytocompatible environment for long‐term spheroid viability (Figure 2c). After thermal crosslinking of the 0.5% dECM bioink at 37°C, its structural properties were characterized by scanning electron microscopy (SEM) to evaluate the pore morphology (Figure 2d). The mean pore size was 111.54±6.31 µm, a dimension sufficient to facilitate effective interactions with the ssDNA‐SWCNT probes (Figure S3). Rheological analyses were conducted to assess its printability and ensure structural stability during the printing process, and confirm the suitability of the 0.5% dECM bioink for in‐bath bioprinting.

Rheological testing confirmed favorable shear‐thinning, Bingham plastic behavior, and shear recovery, ensuring smooth extrusion and post‐printing stability (Figure S4) [32, 33, 45]. Based on the confirmed rheological suitability, the optimized dECM bioink was utilized for spheroid fabrication using both cell‐embedded and bath components (Figure 2e). During the printing process, the selected 0.5% (w/v) formulation supported stable spheroid formation, whereas the 0.25% bioink did not maintain sufficient shape retention for reliable fabrication (Figure S5). Printing pressure was optimized to yield consistent spheroids (∼600 µm diameter) (Figure S6), a size known to induce hypoxia [32, 44]. Hypoxia is critical in TME‐associated epigenetic and phenotypic changes (Figure 2f) [46, 47, 48, 49]. Mimicking such hypoxic conditions is crucial for achieving physiologically relevant screening outcomes. Therefore, the experimental conditions were designed to induce controlled hypoxia within the spheroids. Building upon these optimized parameters, the spheroids were cultured post‐printing for four days to facilitate structural stabilization and maturation. Immunofluorescence for HIF‐1α revealed increasing central fluorescence, confirming the hypoxic core formation over time (Figure 2g; Figure S7a). A concurrent increase in spheroid density likely restricted oxygen diffusion, enhancing hypoxia (Figure S7b) [50, 51, 52]. These findings validate the successful establishment of a physiologically relevant hypoxic microenvironment within the printed spheroids.

### 2.2 Malignancy of 3D Prostate Spheroids

**FIGURE 3:**
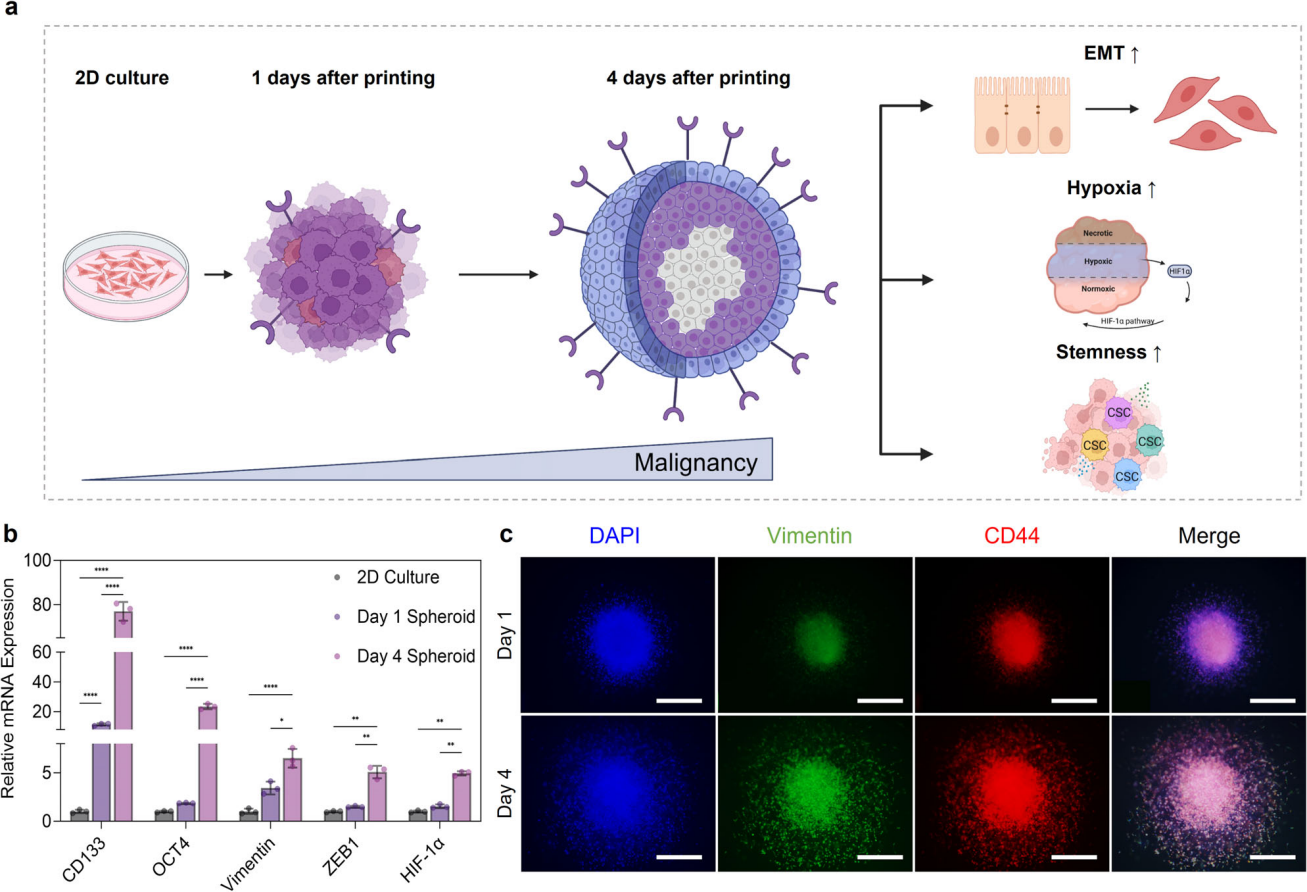
Malignancy of 3D LNCaP spheroids. (a) Schematic illustration depicting time‐dependent malignant progression in spheroids. Over four days of culture, spheroids undergo structural maturation accompanied by epithelial–mesenchymal transition (EMT), hypoxia‐induced HIF‐1α activation, and enrichment of cancer stem cell (CSC) populations. (b) Comparative analysis of relative gene expression of 2D culture, Day 1 spheroid, and Day 4 spheroid. (c) Immunofluorescence staining image results to confirm marker changes in spheroids by day Scale bar, 500 µm. The bar graphs represent the mean values with error bars indicating standard deviation (s.d.). Statistical significance was determined by ordinary one‐way ANOVA using multiple comparisons tests. (^*^ *p* <0.05; ^**^ *p* <0.01; ^***^ *p* <0.001; ^****^ *p* <0.0001). The schematic was produced using BioRender.

Solid tumors are inherently heterogeneous, comprising diverse cancer cell subpopulations with distinct phenotypes and malignant potential. This heterogeneity arises from interactions with the tumor microenvironment, including the oxygen and nutrient gradients, ECM composition, and dynamic cell–cell and cell‐ECM interactions [15, 16, 17]. Recapitulating this heterogeneity in vitro is essential for developing physiologically relevant cancer models. Using optimized materials and printing parameters, 3D LNCaP spheroids were fabricated via in‐bath bioprinting with dECM‐based bioinks. This approach introduces microenvironmental cues that promote phenotypic diversity. The dECM bioink provides ECM‐like biochemical signals that enhance adhesion, proliferation, and differentiation, while the spheroid architecture amplifies cell‐cell contact and mechanical signaling [32, 33, 34, 35, 53, 54]. As spheroids mature, limited oxygen and nutrient diffusion induce hypoxia, activating HIF‐1α and promoting the malignant phenotypes [46, 47, 48, 49]. These conditions contribute to intratumoral heterogeneity, resembling native tumor tissues. The hypothesis is that spheroids generated via in‐bath bioprinting would exhibit enhanced phenotypic heterogeneity compared to conventional 2D cultures (Figure 3a). The 2D cultures, Day 1 spheroids, and Day 4 spheroids were compared to evaluate this. Gene expression analysis revealed the progressive upregulation of stemness markers (CD133, Oct4), EMT markers (vimentin, ZEB1), and HIF‐1α, with the Day 4 spheroids showing the highest levels (Figure 3b). Immunofluorescence confirmed increased CD44 and vimentin expression in Day 4 spheroids (Figure 3c), indicating a shift toward a mesenchymal and aggressive phenotype. Based on these findings, the Day 4 spheroids were selected as the target for the subsequent Cell‐SELEC process. These spheroids represent a hypoxia‐driven, transcriptionally reprogrammed tumor‐like state, enabling aptamer selection against clinically relevant malignant subpopulations under physiologically stressful conditions.

### 2.3 SELEC of ssDNA‐SWCNT Probes With Prostate Cancer Cells

**FIGURE 4:**
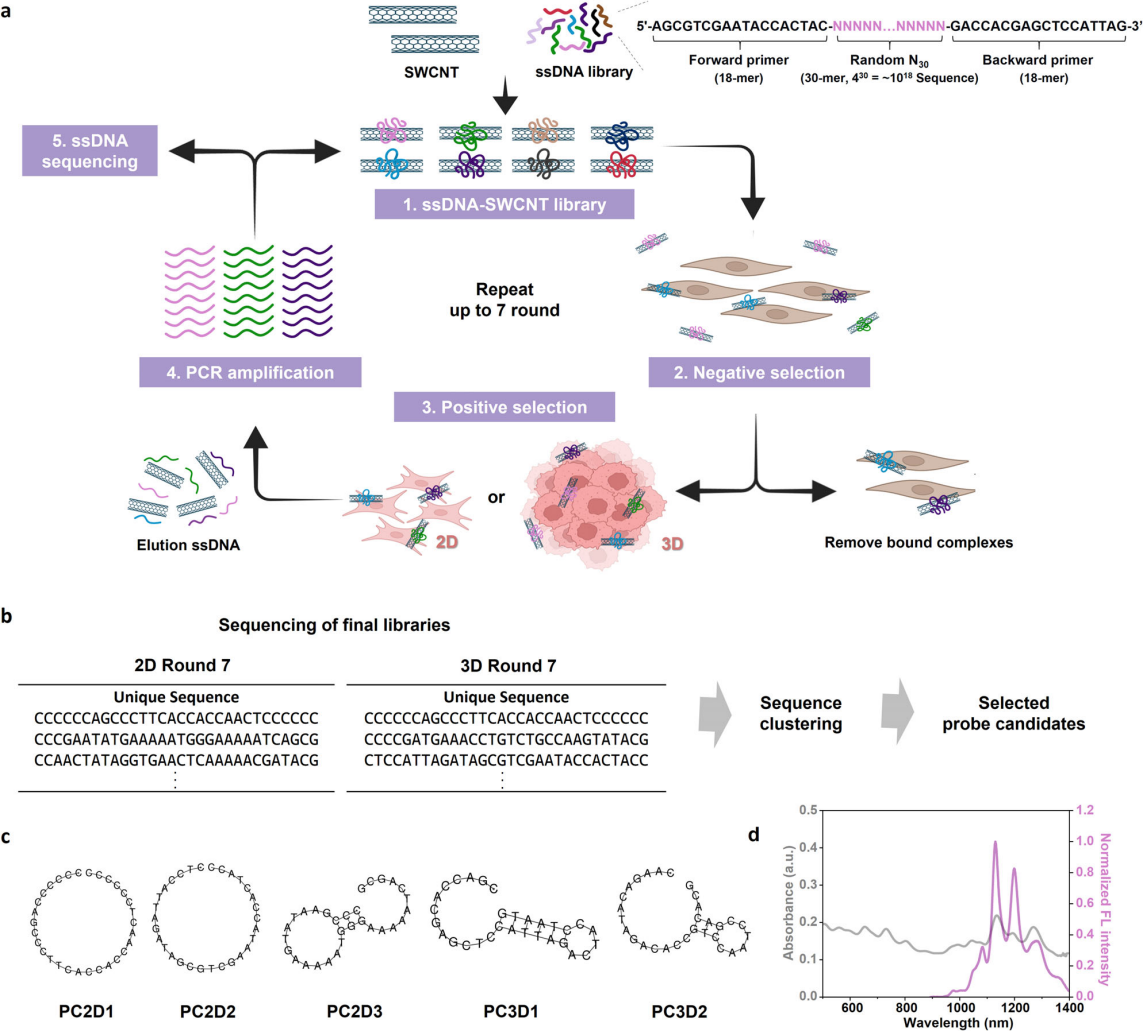
SELEC screening of prostate cancer‐specific ssDNA‐SWCNT probes. (a) Schematic diagram of the SELEC workflow for prostate cancer‐targeting ssDNA‐SWCNT probes. A random ssDNA library with a 30‐nt variable region flanked by 18‐nt primer sites was complexed with HiPco SWCNTs to generate a diverse ssDNA‐SWCNT hybrid library. The library was screened against LNCaP prostate cancer cells through multiple rounds of negative and positive selection under two parallel pathways: 2D monolayer culture and 3D spheroid culture. (b) Workflow for sequence processing and clustering analysis after high‐throughput sequencing. The terminal primer regions were trimmed, and the unique 30‐mer variable regions were ranked by read frequency. Highly similar sequences were grouped into clusters using the AptaSUITE platform. The top‐ranking sequence from each cluster was selected for probe development. (c) Representative secondary structure predictions for the selected ssDNA sequences from the final SELEC round (Round 7) using an online DNA folding tool. The predicted structures include hairpin loops, external loops, and circular motifs, indicating potential conformational diversity prior to SWCNT adsorption. (d) Optical characterization of selected ssDNA‐SWCNT probe. UV–vis–NIR absorption spectra (gray) and normalized NIR‐II fluorescence emission spectra (purple) were measured for the PC3D2‐SWCNT probe at 5 mg/L under 721 nm excitation. The schematic was produced using BioRender.

To identify prostate cancer‐specific ssDNA‐SWCNT probes, we implemented SELEC, a modified high‐throughput SELEX‐based approach (Figure 4a; Figure S8). Previously applied to the development of serotonin sensors [55] and the identification of DNA ligands with strong binding affinity to SWCNT surface [56], SELEC enables the enrichment of ssDNA sequences with high binding and functional capabilities.

A random ssDNA‐SWCNT library was prepared by tip‐sonicating HiPco SWCNTs with the ssDNA pool in PBS buffer. Eachx ssDNA strand contained a 30‐nt randomized region (4 [53, 54] ≈ 1.1 × 10^18^ unique sequences) flanked by 18‐nt primer sites for PCR amplification (Figure 4a). The ssDNA‐SWCNT library was screened against LNCaP prostate cancer cells via two parallel selection pathways: a 3D spheroid model (Pathway 1) and a 2D monolayer culture (Pathway 2). This dual‐track strategy enabled the identification of high‐affinity ssDNA‐SWCNT probes for LNCaP cells under physiologically relevant tumor‐mimicking environments (Pathway 1) while emphasizing negative selection to eliminate non‐specific binding (Pathway 2). Both pathways began with 2D positive selection until Round 4. Pathway 1 then transitioned to 3D spheroids, while Pathway 2 remained in 2D through Round 7. Negative selection was introduced in Round 4 for Pathway 1 and continued through Rounds 4–7 in Pathway 2 (Table S1).

In each round, the ssDNA‐SWCNT library was incubated with LNCaP cells at 4°C on a shaker for 1 h, followed by triple PBS washes to remove the unbound probes. The ssDNA‐SWCNT bound on the membrane of LNCaP cells were isolated at 95°C and amplified using PCR to generate the next‐round ssDNA library. After seven selection rounds, the final ssDNA libraries enriched for high‐binding affinity from both 2D and 3D models were analyzed using high‐throughput sequencing on the Illumina Novaseq 6000 platform. The sequences with high read frequency were shown after trimming the flank primer regions (Figure 4b). Although both pathways yielded enriched candidates, the 3D spheroid model exhibited convergence toward a few dominant sequences in the top ranking, reducing diversity. Clustering analysis was performed to address this using the AptaSUITE open‐source tool (available at https://drivenbyentropy.github.io/) (Figure S9) [57, 58]. From Round 7 libraries, 10 and six clusters were selected from the 2D and 3D pathways, respectively, excluding those with excessively sequence similarities. The most abundant sequence from each cluster was designated as a representative, yielding 16 final candidate probes, denoted as PCND‐M, where PC, N, and M represent prostate cancer, selection condition (2D or 3D), and cluster ranking in descending sequence diversity (Table S2).

For the 16 selected candidate sequences, the secondary structures were predicted using a DNA secondary structure prediction tool (available at https://en.vectorbuilder.com/tool/dna‐secondary‐structure.html), revealing structural motifs such as circular forms, hairpin loops, and external loops (Figure 4c), which would attribute the structural DNA conformations on SWCNT. The optical properties were examined by fabricating, each ssDNA‐SWCNT conjugate using all 16 selected sequences. The absorbance and NIR‐II fluorescence spectra were measured using the PC2D1‐SWCNT (5 mg/L) as a representative (Figure 4d). Optical characterization showed no significant differences in absorption or fluorescence across the 16 probes.

### 2.4 Evaluation of Probes for Targeting LNCaP Cells in 2D Monolayers and 3D Spheroids

**FIGURE 5:**
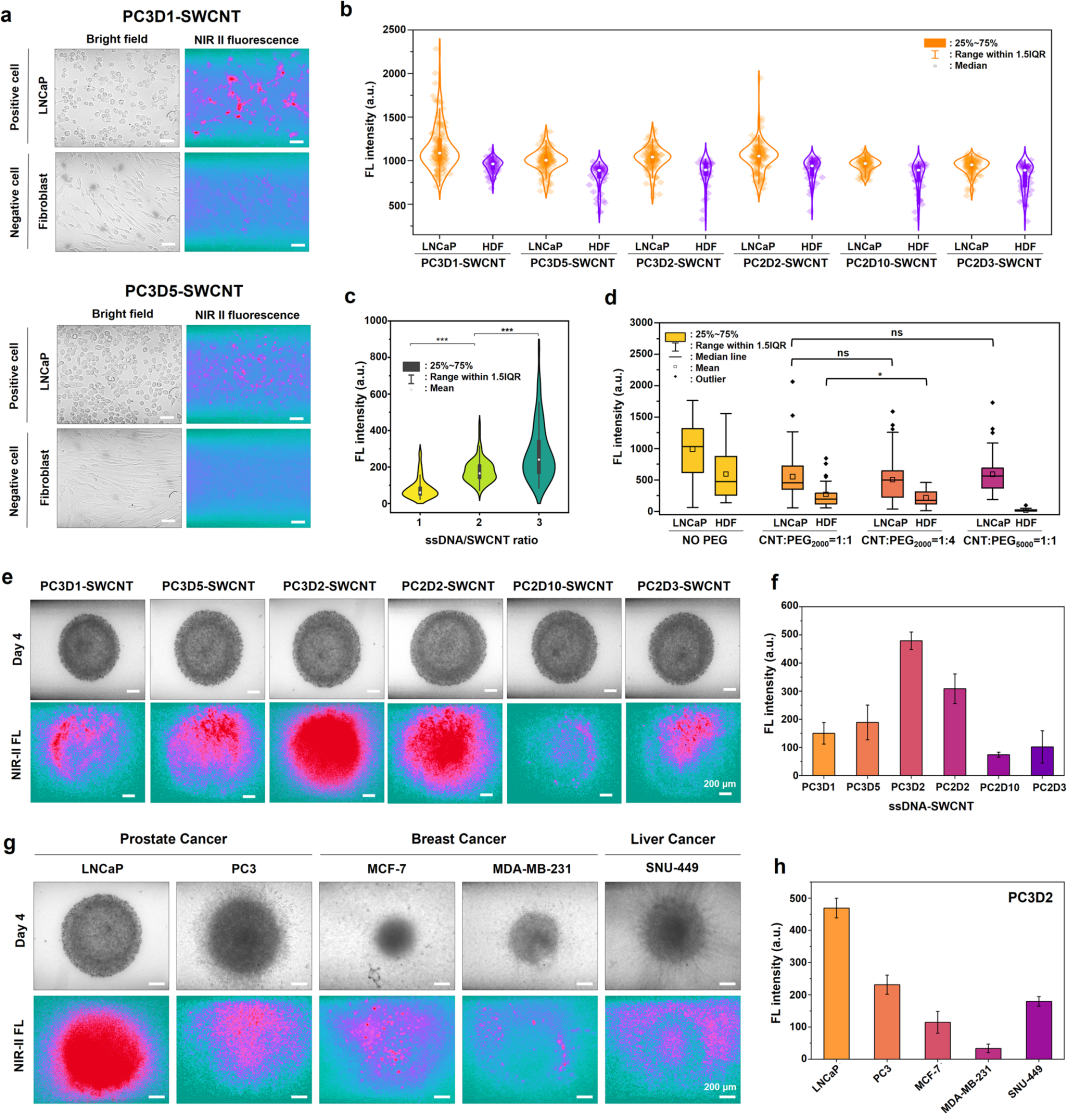
Evaluation of ssDNA‐SWCNT probes for targeting LNCaP cells in 2D cultures and 3D spheroids. (a) Validation of the target efficiency for probes 1 and 2 in 2D cultures. Bright‐field and NIR‐II fluorescence images were obtained in both LNCaP and fibroblast. The scale bar is 20 𝜇m. (b) The distribution of fluorescence intensity in each cell within the field of view (FOV) was compared to evaluate the difference between probes due to variations in target specificity. (c) Improvement in the probe targeting efficiency by increasing the ssDNA/SWCNT mass ratio from 1:1 to 1:3. (d) Enhancement of the probe performance by reducing non‐specific binding by introducing phospholipid‐PEG, along with adjustments to the SWCNT/PEG mass ratio and PEG length. (e) Validation of probe targeting in a 3D spheroid model for improved probes. Bright‐field and NIR‐II fluorescence images were acquired in a 3D spheroid. The scale bar is 200 𝜇m. (f) Quantification of the fluorescence intensity within spheroids in (e). (g) Extension of the probe 3 targeting to the other cancer cell lines, including prostate (LNCaP, PC3), breast (MCF‐7, MDA‐MB‐231), and liver (SNU‐449) cancer cells. (h) Quantification of fluorescence intensity within spheroids in (g).

The specificity of 16 ssDNA‐SWCNT probes was assessed by NIR‐II fluorescence imaging in 2D monolayer cultures using LNCaP cells as the target and HDF as negative controls. Among the tested probes, PC3D1‐SWCNT and PC3D5‐SWCNT showed the most prominent fluorescence contrast, with a strong signal in LNCaP cells and minimal intensity in HDFs, even though PC3D1 exhibited slightly higher non‐specific binding (Figure 5a; Figure S10). The fluorescence intensities from individual cells were quantified, highlighting the top six probes with the greatest signal difference (Figure 5b).

Two optimization strategies were applied to enhance targeting performance. The first approach focused on increasing the ssDNA/SWCNT mass ratio from 1 to 3, resulting in enhanced fluorescence intensity and confirmed probe targeting (Figure 5c). The second strategy reduced non‐specific binding by modifying the ssDNA‐SWCNTs with phospholipid‐polyethylene glycol (PL‐PEG) via noncovalent interactions, optimizing the PEG/SWCNT mass ratio and the PEG chain length. At a 1:1 SWCNT:PEG2000 ratio, the fluorescence decreased more in HDFs (2.2‐fold) than in LNCaP cells (1.7‐fold), indicating improved specificity (Figure 5d). Increasing the PEG/SWCNT ratio to 4:1 reduced fluorescence further in HDFs (1.8‐fold decrease) with a minimal effect on LNCaP cells (1.09‐fold decrease). Furthermore, extending the PEG chain length to PEG5000 at a 1:1 PEG/SWCCNT led to a 17.3‐fold decrease in non‐specific binding in HDFs compared to PEG2000, emphasizing the role of the PEG chain length in enhancing the probe specificity. Optimization studies identified PL‐PEG5000/ssDNA‐SWCNT as the optimal probe configuration, with SWCNT:ssDNA:PL‐PEG mass ratio of 1:3:1, applied at 20 mg/L with incubation in 2D cultures for 1 min. NIR‐II imaging confirmed that the optimized probes exhibited enhanced specificity and reduced background fluorescence significantly compared to previous probes (Figure S11).

The binding affinity was quantified via a cell‐based binding affinity assay using NIR‐II imaging. The LNCaP cells were incubated with various concentrations (100 pM to 500 nM) of PC3D1 and PC3D2/PL‐PEG‐SWCNT probes. Equilibrium was reached by 25 min. Hill fitting yielded K_d_ values of 21 and 14 nM for PC3D1 and PC3D2, respectively, indicating high‐affinity interactions with cell surface targets (Figure S12).

The targeting efficiency was also evaluated in 3D LNCaP spheroids in the top six probes. The 1‐day and 4‐day spheroids were treated with 50 mg/L of probes for 10 min, with probes PC3D2 and PC2D2/PL‐PEG‐SWCNT generating strong, evenly distributed NIR‐II fluorescence (Figure 5e). Fluorescence quantification showed that PC3D2 exhibited signals 2‐ to 6‐fold higher than most other probes, except for PC2D2 (Figure 5f). PC3D1 ranked highest in 2D but showed a diminished signal in 3D, suggesting potential microenvironment‐dependent effects. The probes were applied to spheroids from additional cancer cell lines (PC3 (prostate), MCF‐7, and MDA‐MB‐231 (breast), and SNU‐449 (liver)) under identical conditions to determine if PC3D2/PL‐PEG‐SWCNTs selectively target prostate cancer cells or have broader targeting potential. The probes exhibited the highest fluorescence signal (more than two times) in LNCaP spheroids, while also generating detectable fluorescence in the other cancer cell lines (Figure 5g). Quantitative analysis showed relatively high fluorescence in PC3, whereas MCF‐7 and MDA‐MB‐231 spheroids exhibited markedly lower fluorescence levels (Figure 5h). This suggests the moderate specificity of PC3D2 for prostate cancer, with some cross‐reactivity. In contrast, PC2D2/PL‐PEG‐SWCNT produced the strongest signal in LNCaP but much lower fluorescence in other cell lines, suggesting higher LNCaP selectivity (Figure S13). To further validate whether the observed selectivity extends beyond fibroblast controls to normal prostate epithelial cells, additional 2D monolayer experiments were performed using RWPE‐1 cells as a normal prostate cell type (Figure S14). Upon incubation with PC3D2/PEG‐SWCNTs (20 mg/L, 7 min), negligible fluorescence was detected in RWPE‐1 cells, comparable to PC3 cells, whereas LNCaP cells retained substantially higher NIR‐II fluorescence intensity. These results indicate that PC3D2/PEG‐SWCNTs preferentially interact with aggressive prostate cancer cells rather than normal prostate epithelial cells, reinforcing their cell‐type‐specific targeting capability.

### 2.5 Cellular Uptake Study of Probes

**FIGURE 6:**
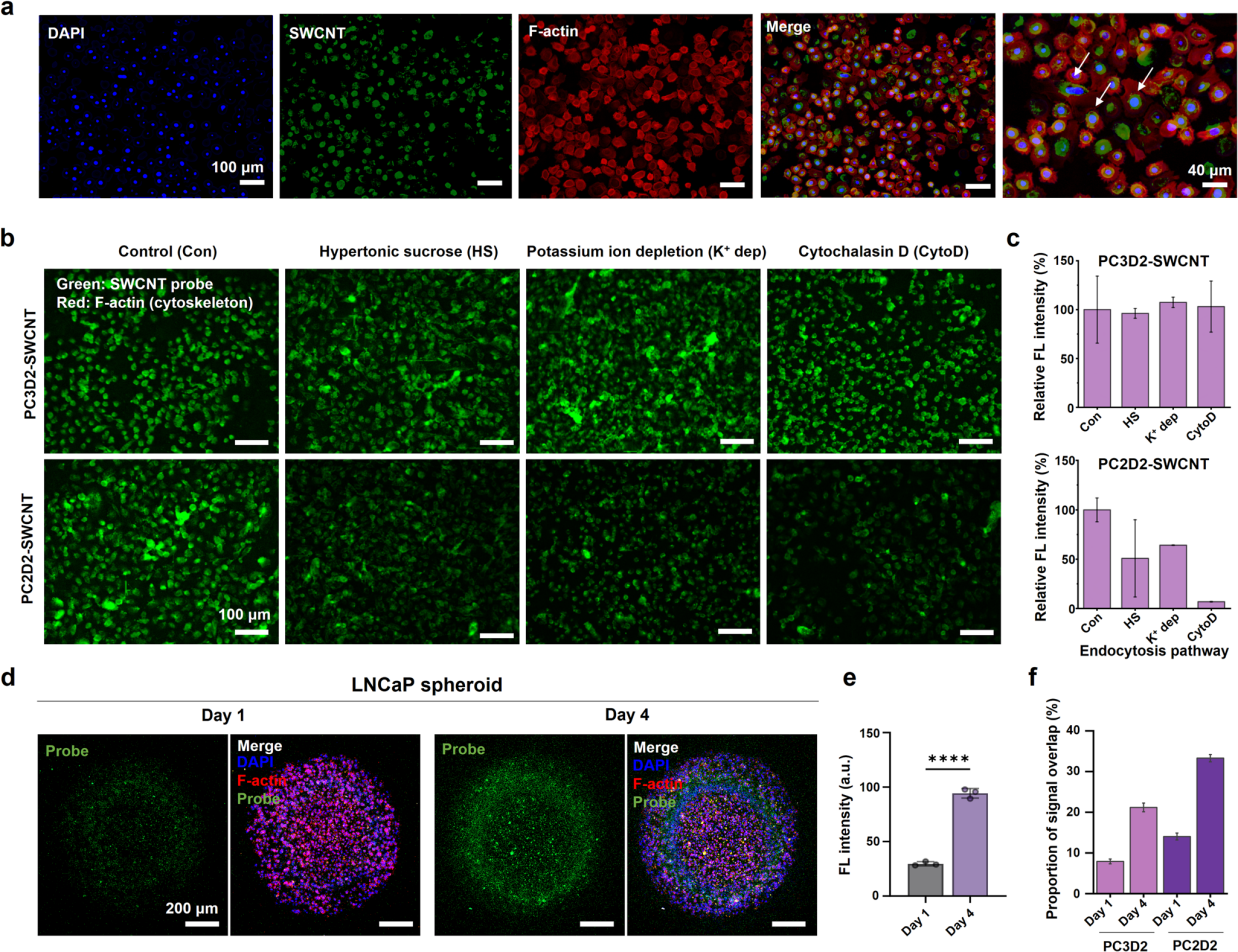
Cellular uptake of ssDNA‐SWCNT probes in LNCaP cells and spheroids. (a) Confocal images showing probe internalization and colocalization with F‐actin and DAPI in LNCaP spheroids on days 1 and 4. The green fluorescence represents FAM‐labeled ssDNA‐SWCNT probes; the red fluorescence marks F‐actin (cytoskeletal organization); blue fluorescence indicates DAPI (nuclear staining). The white arrows indicate the cellular uptake. (b) The investigation of endocytosis of PC3D2 and PC2D2‐SWCNT using inhibitor such as hypertonic sucrose, potassium ion depletion, and cytochalasin D. Hypertonic sucrose and potassium ion depletion inhibit clathrin‐mediated endocytosis, while cytochalasin D inhibits phagocytosis and macropinocytosis. (c) Quantitative analysis of cellular uptake for PC3D2 and PC2D2‐SWCNT. (d) The fluorescence images were stained with DAPI, 20 mg/L of probes, and F‐actin. The confocal data showed that the probe's fluorescence predominantly colocalized with F‐actin in the cytoplasm, rather than with DAPI in the nucleus. (e) Quantification of PC3D2‐SWCNT fluorescence in spheroids on days 1 and 4 showed a significant increase in fluorescence intensity over time. (f) Proportion of signal overlap between the probe and F‐actin. A significant increase in signal overlap was observed for PC3D2 and PC2D2‐SWCNT between days 1 and 4. This enhancement in colocalization with the F‐actin suggests a progressive increase in the cellular uptake of the SWCNT probes.

The uptake of the probes in LNCaP cells was analyzed to evaluate whether they were internalized or remained surface‐bound. In the 2D monolayers, the cells were treated with 20 mg/L of PC3D1‐SWCNT for 10 min and stained with DAPI and F‐actin to visualize the nuclei and cytoskeleton. Because SWCNTs emit only in the NIR‐II range, FAM‐labeled (ACG)_6_ ssDNA was used to enable fluorescence detection, co‐functionalized with PL‐PEG at a 7:3 mass ratio with the target ssDNA. The imaging results showed membrane adsorption and cytoplasmic internalization of the probes (Figure 6a).

The major endocytic pathways, including clathrin‐mediated endocytosis, phagocytosis, and micropinocytosis, were inhibited selectively to elucidate the internalization mechanism. Clathrin‐mediated endocytosis was blocked using hypertonic sucrose [59] and potassium ion depletion [60], while the actin‐dependent pathways (phagocytosis and micropinocytosis) were inhibited with cytochalasin D [61]. The cells were pre‐incubated with these inhibitors at 37°C for 30 min, followed by exposure to 20 mg/L of the probes for 10 min. Epifluorescence imaging from FAM in the probes revealed differential uptake profiles between probe types (Figure 6b; Figure S15). In particular, FAM‐PC3D2/PL‐PEG‐SWCNT showed robust fluorescence signals regardless of pathway inhibition, suggesting that this probe remains surface‐bound or internalizes alternative cellular entry mechanisms. This observation was supported by quantitative fluorescence intensity measurements (Figure 6c). In contrast, FAM‐PC2D2/PL‐PEG‐SWCNT reduced the fluorescence after pathway inhibition, particularly upon cytochalasin D, implicating phagocytosis and micropinocytosis. Additional suppression under hypertonic condition suggested partial reliance on clathrin‐mediated uptake. Quantitative trends are consistent with the imaging results and suggest that the PC2D2 probes are internalized via receptor‐mediated and non‐specific endocytic pathways.

The distinct cellular behaviors of PC3D2 and PC2D2 underscore the critical role of DNA sequence in governing probe–cell interactions. High‐resolution confocal Z‐stack imaging of LNCaP cells revealed that PC2D2/SWCNT probes undergo efficient intracellular uptake, whereas PC3D2/SWCNT probes predominantly remain localized at or near the cell surface (Figure S16). Pharmacological inhibition studies further indicate that PC2D2 internalization occurs via multiple energy‐dependent endocytic pathways, including clathrin‐mediated endocytosis, macropinocytosis, and phagocytosis (Figure 6d).

Although the precise surface receptors responsible for these behaviors remain unidentified, we hypothesize that sequence‐dependent interactions between ssDNA and membrane‐associated proteins, glycoproteins, or lipid domains [62, 63]. initiate either active internalization (PC2D2) or stable surface adsorption without uptake (PC3D2). Such sequence‐specific conformations may mimic motifs recognized by cell‐surface receptors or extracellular matrix components, consistent with previous reports of ssDNA interactions with cell‐surface nucleolin and related receptors [63]. Future affinity‐based proteomic studies will be required to elucidate the molecular targets underlying these differential uptake mechanisms.

To investigate uptake efficiency, confocal microscopy was performed on 3D spheroids treated with FAM‐ssDNA/PL‐PEG‐SWCNT on day 1 and 4 (Figure S17). The fluorescence intensity increased 3‐fold on day 4 than day 1, suggesting that PC3D2 targets a biomarker upregulated during spheroid maturation (Figure 6d,e). 3D confocal analysis revealed that the colocalization of SWCNT with F‐actin in the cytoplasm, indicating >20% probe internalization in day 4 spheroids (Figure 6f). To evaluate the stability of ssDNA‐SWCNT probes under biologically relevant conditions, the dispersive stability of PC3D2 and PC2D2/PL‐PEG‐SWCNT constructs was examined in human serum (1%–20%) at 37°C. Across all serum concentrations and incubation times (∼24 h), the probes maintained overall dispersive stability, with only minor wall‐associated accumulation observed after 6 h and no detectable bulk aggregation or precipitation (Figure S18). This serum‐independent stability suggests that the ssDNA‐SWCNT probes maintain their structural integrity in physiological fluids, supporting their suitability for in vivo applications. The probe cytotoxicity was assessed via live/dead staining (Figure S19). The LNCaP cells were exposed to various concentrations of the probe up to 80 mg/L. The cell viability remained above 98% at 10 min and >90% after 12 h, even at the highest concentration, indicating minimal cytotoxic effects under experimental conditions.

### 2.6 Specific Targeting Capability of ssDNA‐SWCNT Probes in 3D LNCaP Spheroids

**FIGURE 7:**
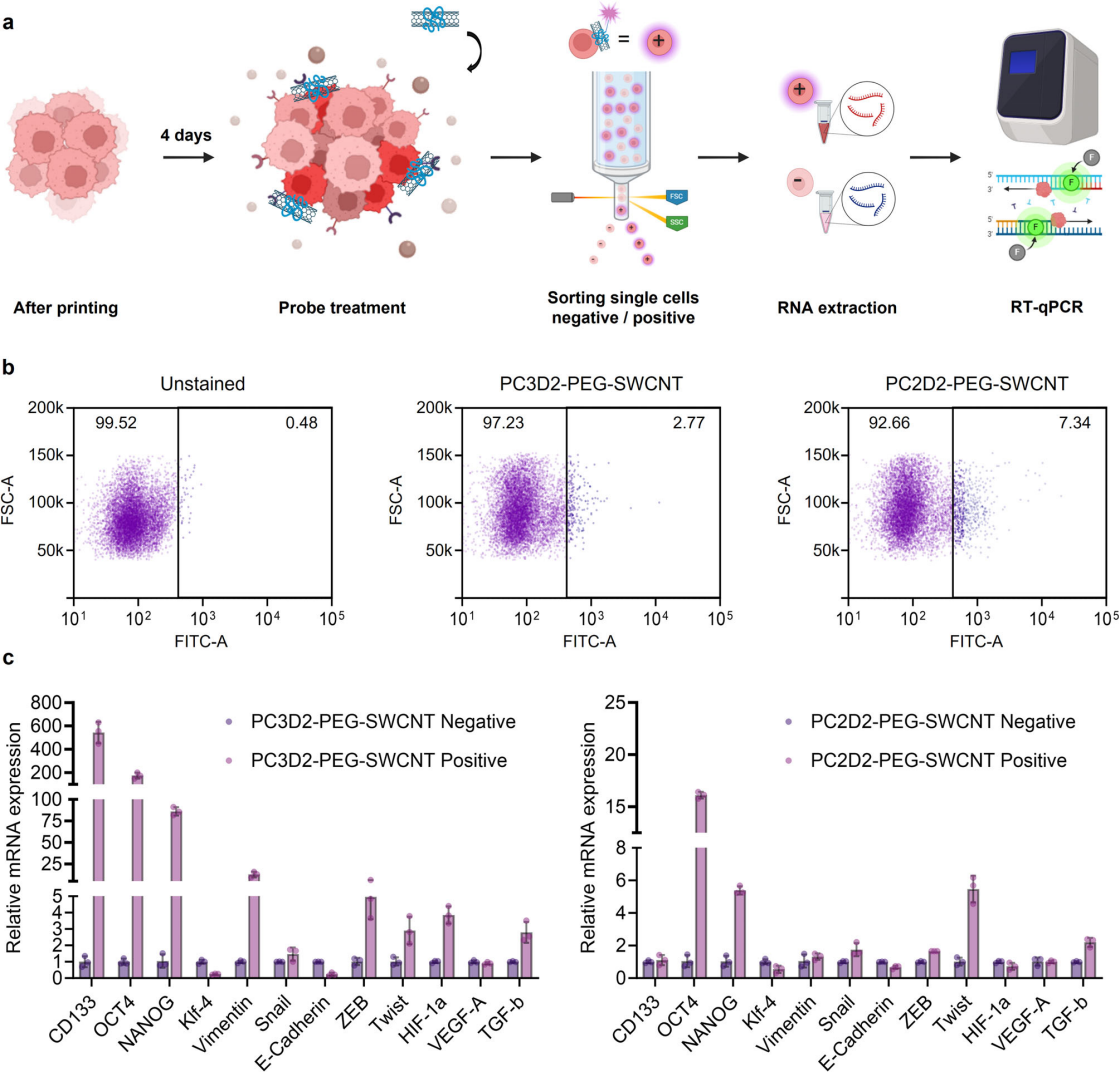
Specificity Analysis of ssDNA‐SWCNT Probes in 3D LNCaP Spheroids. (a) Workflow of cell sorting based on SWCNT‐positive and ‐negative populations, followed by characterization and gene expression analysis using RT‐qPCR. (b) FACS dot plots of SWCNT‐positive and negative cells incubated with PC3D2‐PEG‐SWCNT, PC2D2‐PEG‐SWCNT, and without probe in 4 days LNCaP 3D spheroids. (c) Relative gene expression level of SWCNT‐positive and SWCNT‐negative cells treated with PC3D2‐PEG‐SWCNT and PC2D2‐PEG‐SWCNT probe. Fold changes in the mRNA levels of cancer‐ and stemness‐related genes in SWCNT‐positive cells compared to the corresponding SWCNT‐negative cells for each probe. The gene expression levels were quantified using the double delta Ct analysis method and normalized to the SWCNT‐negative population. The schematic was produced using BioRender.

LNCaP spheroids were cultured for 4 days to facilitate structural maturation and establish a physiologically relevant tumor microenvironment. After maturation, two ssDNA–SWCNT probes (PC3D2/PL‐PEG‐SWCNT and PC2D2/PL‐PEG‐SWCNT were applied to the spheroids to evaluate their molecular binding profiles within the 3D context. For fluorescence‐activated cell sorting (FACS), the FAM‐conjugated ssDNAs were co‐adsorbed onto each probe for green fluorescence detection. Probe incubation, FACS‐based separation, RNA extraction, and RT‐qPCR‐based gene expression analysis were performed sequentially (Figure 7a). Probe‐positive and ‐negative cell populations were separated by FACS based on the fluorescence intensity. A FAM‐labeled random N30 DNA‐SWCNT was also used as the control group. A comparative heatmap analysis of gene expression across the N30 DNA‐SWCNT and PC3D2 probe groups revealed clear differences in molecular signatures. Notably, PC3D2‐PEG‐SWCNT‐Positive exhibited selective upregulation of the key stemness‐ and EMT‐related markers including CD133, OCT4, Vimentin, and HIF‐1α compared to the N30‐SWCNT‐Negative, N30‐SWCNT‐Positive, and PC3D2‐PEG‐SWCNT‐Negative groups. These differences further support the selective interaction of the probe with a distinct subpopulation within the spheroid model. (Figure S20). Next, the molecular characteristics of the subpopulations targeted were compared by PC3D2/PL‐PEG‐SWCNT and PC2D2/PL‐PEG‐SWCNT, with their respective probe‐negative populations serving as the controls. The PC2D2/PL‐PEG‐SWCNT and PC3D2/PL‐PEG‐SWCNT probes exhibited 7.34% and 2.77% probe‐positive cell populations, respectively (Figure 7b). Subsequent gene expression profiling revealed that PC3D2 and PC2D2 target phenotypically distinct subpopulations (Figure 7c). The PC3D2/PL‐PEG‐SWCNT‐positive cells showed striking upregulation of the stemness markers: CD133 (628‐fold), OCT4 (201‐fold), and NANOG (91‐fold). In parallel, EMT‐associated genes such as Vimentin (9.3‐fold), ZEB (6.3‐fold), and Twist (3.7‐fold) were markedly elevated, with a strong reduction in E‐cadherin, indicating progression toward a mesenchymal phenotype [64, 65, 66]. Specifically, HIF‐1α was also increased (3.3‐fold), which is consistent with previous observations in 3D spheroids. In contrast, the PC2D2/PL‐PEG‐SWCNT‐positive cells exhibited more moderate increases in OCT4 (15.7‐fold) and NANOG (5.2‐fold), with no significant change in CD133. EMT markers such as Twist (6.1‐fold), ZEB (1.6‐fold), and Vimentin (1.3‐fold) were elevated alongside the downregulation of E‐cadherin, suggesting a mesenchymal‐shifted profile. Unlike PC3D2, PC2D2 did not show significant HIF‐1α upregulation, indicating minimal hypoxic association. This highlights their selective binding within the spheroid and contrasts with the minimal differences observed between positive and negative populations in the N30 DNA‐SWCNT group. Furthermore, expression of TGF‐β1, a common regulator of both stemness and EMT, was increased in both groups (3.5‐fold and 2.3‐fold in PC3D2 and PC2D2, respectively). Interestingly, KLF4 expression was reduced in both probe‐positive populations compared to their respective negative controls. Previous studies have reported that KLF4 functions as a tumor suppressor in prostate cancer [67, 68, 69], contributing to the inhibition of tumor cell proliferation and migration. On the other hand, its downregulation has been associated with increased tumor aggressiveness and progression. The observed reduction in KLF4 expression in both probe‐positive groups may suggest that the targeted subpopulations are associated with a more aggressive phenotype. These results define two phenotypically distinct probe‐binding populations: PC3D2 targets a stem‐like, hypoxia‐adapted, and EMT‐progressive subpopulation, whereas PC2D2 labels a mesenchymal, partially stem‐like population lacking a hypoxic profile. These findings support the differential molecular specificity of each probe within the heterogeneous 3D spheroid environment.

### 2.7 Drug Delivery of ssDNA‐SWCNT Probes

**FIGURE 8:**
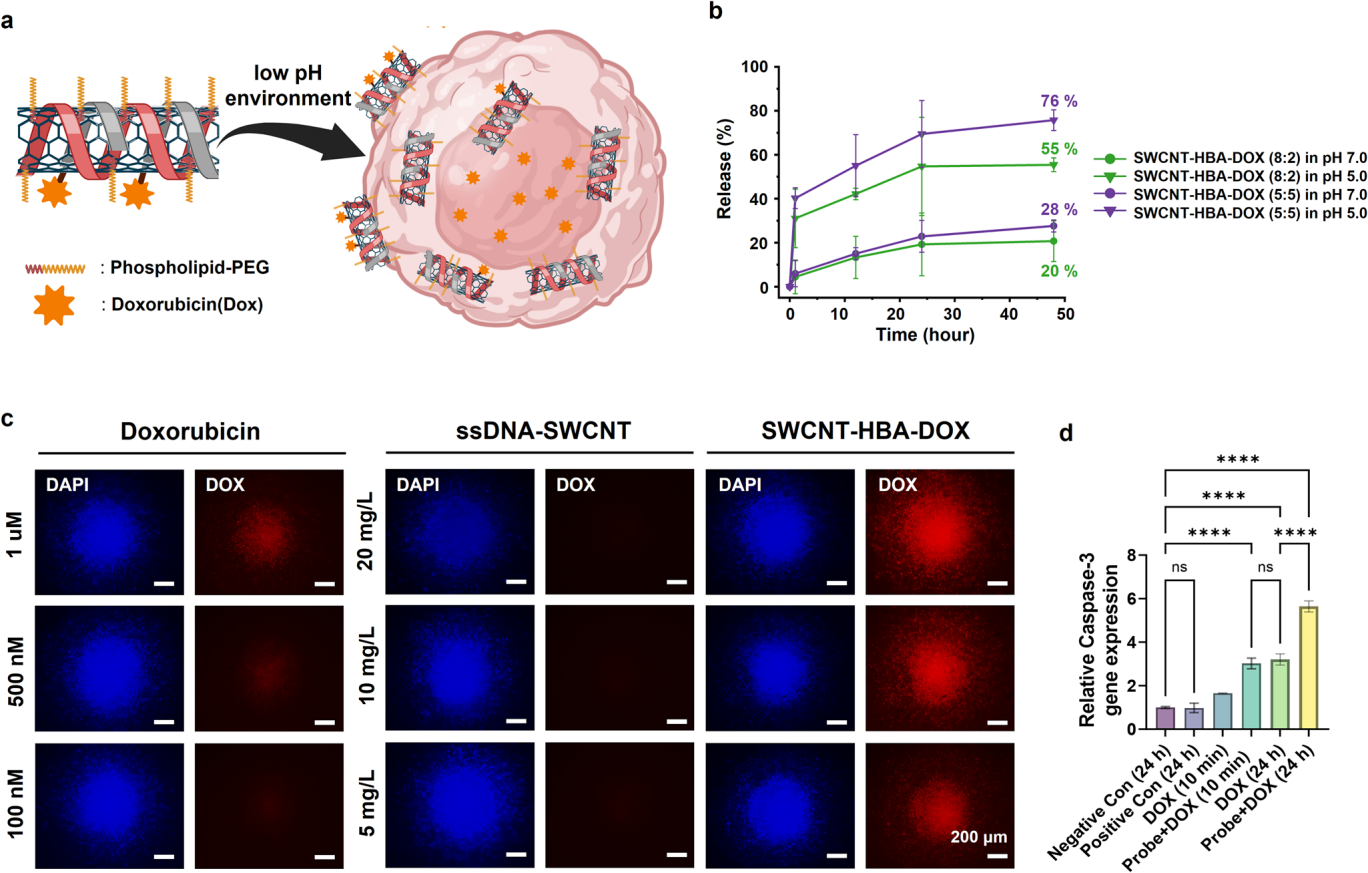
Drug delivery of ssDNA‐SWCNT probes. (a) Schematic illustration of ssDNA‐SWCNT probes conjugated with doxorubicin, enabling pH‐responsive drug release. (b) Quantification of doxorubicin release (%) from ssDNA‐SWCNT probes over time at different pH conditions. DOX release was evaluated at pH 7.0 and pH 5.0 by absorbance at 490 nm. The release efficiency depended on the target ssDNA:NH_2_‐(ACG)_6_ ssDNA ratio in SWCNT‐DOX/PL‐PEG probes (8:2 vs. 5:5). (c) Fluorescence microscopy images showing doxorubicin localization (red channel) after 24‐h treatment with different formulations: Doxorubicin only, ssDNA‐SWCNT only, and SWCNT‐HBA‐DOX at various concentrations. (d) RT‐qPCR analysis of relative caspase‐3 gene expression across different groups after drug treatment and washing, followed by a 48‐h incubation period. The bar graphs represent the mean values with error bars indicating standard deviation (s.d.). Statistical significance was determined by ordinary one‐way ANOVA using multiple comparisons tests. (^*^ *p* <0.05; ^**^ *p* <0.01; ^***^ *p* <0.001; ^****^ *p* <0.0001). The schematic was produced using BioRender.

The SWCNT platform was extended to therapeutic applications by engineering a pH‐responsive doxorubicin (DOX) delivery system for targeted drug release in the acidic tumor microenvironment. The SWCNTs were functionalized with PC2D2 (target‐recognizing ssDNA) and NH_2_‐(ACG)_6_ sequences, the latter enabling DOX conjugation via hydrazinobenzoic acid (HBA), forming acid‐labile hydrazone bonds (Figure 8a) [70, 71].

The drug‐release efficiency was evaluated by assessing the in vitro DOX release profiles at pH 7.0 (physiological conditions) and pH 5.0 (tumor‐mimicking acidic conditions) using absorbance at 490 nm (Figure S21). The DOX release efficiency varied according to the target: NH_2_‐(ACG)_6_ ssDNA ratio (8:2 vs. 5:5) in the SWCNT‐HBA‐DOX with PL‐PEG. At pH 7.0, the 8:2 probe exhibited 20% DOX release, while the release increased to 55% at pH 5.0. In contrast, the 5:5 probe showed a higher release efficiency, with 28% and 76% at pH 7.0 and 5.0, respectively, indicating enhanced DOX release under acidic conditions compared to the 8:2 formulation (Figure 8b).

To assess drug delivery and therapeutic efficacy, 3D prostate cancer spheroids were incubated for 24 h with varying concentrations of SWCNT‐HBA‐DOX (5, 10, and 20 mg/L), alongside free DOX and ssDNA‐SWCNT without DOX. Based on estimated DOX loading efficiency, the corresponding loaded DOX concentrations in the SWCNT‐HBA‐DOX solutions were 375 nM, 750 nM, and 1.5 µM, respectively. The fluorescence imaging of DOX emission confirmed that SWCNT‐DOX conjugates at 5 mg/L exhibited a 2.2‐fold increase in targeting efficiency compared to free DOX (500 nM), suggesting that the nanocarrier effectively facilitated intracellular DOX delivery (Figure 8c).

The therapeutic efficacy of the system was evaluated further by measuring relative Caspase‐3 gene expression, a key marker of apoptosis. The results revealed that SWCNT‐HBA‐DOX conjugates significantly enhanced apoptosis over time, particularly after 24 h of treatment, compared to free DOX (Figure 8d). In particular, treatment with 5 mg/L of SWCNT‐HBA‐DOX resulted in a 1.8‐fold increase in Caspase‐3 expression compared to 375 nM free DOX, having the same DOX concentration. Hence, SWCNT‐HBA‐DOX delivery enhances intracellular drug accumulation and retention, leading to a more potent apoptotic response. These findings demonstrate that SWCNT‐HBA‐DOX conjugates represent a promising nanoplatform for targeted prostate cancer therapy, offering enhanced drug delivery efficiency, selective pH‐responsive release, and superior anticancer activity.

To examine whether additional surface functionalization for drug delivery affects cellular targeting specificity, the fluorescence responses of drug‐loaded PC2D2/SWCNT‐HBA‐DOX were compared with those of unloaded PC2D2/SWCNT probes in normal prostate epithelial RWPE‐1 cells (Figure S22). Both probes exhibited only minimal fluorescence in RWPE‐1 cells, indicating negligible nonspecific uptake following drug loading. In contrast, free doxorubicin showed strong fluorescence in RWPE‐1 cells, confirming its nonspecific cellular accumulation. These results demonstrate that the additional surface chemistry introduced for drug delivery does not compromise the sequence‐dependent targeting specificity of the DNA/SWCNT probes.

## 3 Discussion

In this study, we established a high‐throughput SELEC platform for identifying ssDNA‐functionalized SWCNT probes targeting prostate cancer cells, particularly within 3D spheroid models that recapitulate the tumor microenvironment. By screening a large ssDNA library (∼10^1^ ^8^ sequences) and integrating NIR‐II imaging, probe optimization, and binding affinity analysis, high‐affinity probes with selective targeting capabilities under physiologically relevant conditions were identified.

A distinct advantage of the proposed SELEC approach is the ability to simultaneously identify a diverse repertoire of functional probes from an immense ssDNA library. This differs from the conventional 1:1 strategy, where one probe is engineered, evolved, or optimized for a single, predefined molecular target. In contrast, the platform enables the multiplexed discovery of candidate probes that bind to diverse cellular subpopulations within complex biological samples through a single iterative selection experiment. Through the corona phase recognition mechanism, the ssDNA wrapping conformation produces a unique molecular interface that can selectively interact with protein epitopes and other classes of biomolecules, including lipids, glycans, and metabolite‐related surface moieties. This allows the identification of probes that recognize composite molecular features related to cell phenotypes, without requiring prior knowledge of specific biomarkers.

Although aptamers have been widely used for molecular recognition, aptamer conformation can be disrupted when adsorbed onto the nanomaterial surface, leading to a loss of target‐binding efficiency. In support of this concern, Landry et al. evaluated a library of nine SWCNT–aptamer sensor constructs and reported that only two retained strong and selective fluorescence responses to their respective target proteins, RAP1 and HIV1 integrase [72]. This outcome underscores the conformational fragility of many aptamers when interfaced with SWCNTs, and the need to consider surface‐induced conformational changes during sensor design. These findings reinforce the rationale for using a high‐throughput selection strategy based on a random ssDNA library adsorbed onto SWCNTs initially. This approach enables the discovery of sequences that retain target affinity while constrained on the nanotube surface. By directly selecting for molecular recognition under the physical constraints of the SWCNT corona phase, the method ensures compatibility between the recognition function and nanoscale surface conformation, which is critical for sensor performance in complex biological environments.

Based on the successful high‐throughput screening of ssDNA‐SWCNT probes, this study emphasizes the methodological strength and broader implications of the SELEC approach. Therefore, different ssDNA‐SWCNT probes can target distinct biomarker‐expressing subpopulations within heterogeneous LNCaP spheroids. These results demonstrate the two ssDNA‐SWCNT probes (PC2D2 and PC3D2) have phenotype‐specific targeting affinity for distinct biomarker‐expressing subpopulations within heterogeneous LNCaP spheroids. Corona phases (consisting of ssDNA tertiary structure on the SWCNT surface) of two probes appear to interact with distinct surface features on the cellular subpopulation. PC3D2, selected using a 3D Cell‐SELEC strategy using a spheroid, bound to a subpopulation characterized by the strong expression of stemness markers such as CD133, OCT4, and NANOG, along with EMT regulators including Vimentin, ZEB, and Twist. E‐cadherin expression was reduced concomitantly, indicating a transition toward a mesenchymal phenotype. Notably, HIF‐1α was also upregulated in this group, suggesting an adaptation to the hypoxic condition inside the spheroid core. In contrast, PC2D2‐positive cells, selected through a 2D SELEC strategy, exhibited similar EMT‐related changes but did not show increased CD133 or HIF‐1α expression, suggesting a less stem‐like and non‐hypoxic subpopulation.

The elevated expression of HIF‐1α in PC3D2‐bound cells is consistent with increased HIF‐1α levels within the spheroid core (Figure 2h,i). It implies that the 3D SELEC process enriched the probes targeting hypoxia‐adapted cells conditioned by the 3D microenvironment. Furthermore, the strong increase in CD133 observed in the PC3D2‐positive population aligns with previous findings in that hypoxic stress can induce CD133 expression through HIF‐1α–mediated transcriptional activation in LNCaP cells [73]. Consistently, HIF‐1α has been shown to increase the CD133^+^ population and promote sphere formation, whereas silencing HIF‐1α reduces stem‐like features in prostate cancer cells [74]. HIF‐1α has also been reported to activate the CD133 promoter through cooperation with ETS family transcription factors, supporting its role as a molecular regulator of stemness [75, 76, 77]. These findings suggest that PC3D2 prefers the subpopulation under hypoxia, stemness, and spatial adaptation within the 3D tumor microenvironment. In particular, the divergent cellular uptake mechanism and fluorescence intensities associated with different probes (Figure 6b,c) suggest that these nanostructures can distinguish subpopulations based on their biomarker composition, offering a powerful strategy for mapping tumor heterogeneity.

From a translational perspective, it is also important to consider the biological safety and compatibility of the ssDNA‐SWCNT probes. In this study, live/dead staining assays demonstrated minimal cytotoxic effects in LNCaP cells, even at relatively high probe concentrations and extended incubation times (Figure S19). While a comprehensive evaluation of immunogenicity was beyond the scope of this work, the PL‐PEG surface passivation strategy employed here has been widely reported to reduce immune recognition and improve biocompatibility in PEGylated SWCNT systems [78, 79, 80]. Previous studies have shown low immunotoxicity and favorable in vivo behavior for similar PEGylated CNT constructs, suggesting that the surface‐engineered ssDNA‐SWCNT probes used in this study may be suitable for biological and translational applications. These considerations highlight the potential of the platform for future in vivo studies, while emphasizing the need for dedicated immunological and pharmacokinetic evaluations.

The ssDNA‐SWCNT constructs serve as targeting ligands and imaging moieties, owing to the molecular recognition capabilities of the corona phase and the intrinsic NIR‐II fluorescence of SWCNTs. This dual functionality enables the real‐time visualization of molecular interactions and cellular uptake, while simultaneously providing target specificity. Integrating targeting and imaging within a single nanostructure simplifies system design, improves spatiotemporal resolution, and eliminates the need for external labels, making it well‐suited for image‐guided diagnostics and therapy. ssDNA‐SWCNT probes are more cost‐effective, stable, and easily multiplexed than antibodies. Considering the lateral dimension of SWCNT (>300 nm) and ssDNA (∼3 nm), dozens of multiple functional ssDNA sequences can be co‐adsorbed onto a single SWCNT, enabling multivalent interactions with several biomarkers simultaneously. This multivalency enhances the binding affinity and enables more comprehensive engagement with complex tumor microenvironments. The ability to target a range of biomarkers further provides the utility of this platform in addressing tumor heterogeneity, a key challenge in cancer diagnosis and treatment. Single‐biomarker strategies often fail to capture the complexity of the tumor. ssDNA‐SWCNT probes enable a more comprehensive analysis of the tumor microenvironment and offer a route to overcoming the limitations of monotherapy by leveraging multiplexed targeting.

A direct comparison between SELEC‐selected probes and randomly chosen DNA sequences provides important insight into the origin of probe selectivity. Randomly selected five DNA/SWCNT constructs exhibited fluorescence signals close to background levels in LNCaP cells, indicating minimal cellular accumulation and confirming that nonspecific DNA wrapping alone is insufficient to achieve effective targeting (Figures S23 and S24). In contrast, the strong and selective fluorescence observed for PC3D2 highlights that meaningful probe–cell interactions arise from sequence‐dependent selection. These observations suggest that functionally potent probes originate from rare DNA sequences that form favorable corona‐phase conformations on the SWCNT surface. Accordingly, reliance on low‐throughput, arbitrary sequence testing is likely to overlook such rare but effective candidates, underscoring the necessity of systematic and scalable high‐throughput screening strategies, such as the SELEC platform employed in this study.

In future studies, the SELEC method can be extended for in vivo tumor model, such as xenograft or syngeneic mouse models, where the evolution protocol would be adapted to enrich the tumor‐targeting ssDNA‐SWCNT probes following intravenous administration. This in vivo selection approach would enable the identification of probes with enhanced tumor accumulation and retention under physiologically relevant conditions. In addition, critical factors influencing probe performance in vivo, including non‐specific biodistribution to off‐target organs and the formation of protein corona on the nanoparticle surface upon exposure to serum proteins, need further investigation. Understanding these parameters will be important for optimizing probe design and maximizing tumor‐targeting specificity while minimizing systemic clearance.

In conclusion, this study underscores the versatility of the ssDNA‐SWCNT platform as a highly selective cancer cell recognition tool and as a tunable nanocarrier system with the potential for multivalent targeting and controlled drug delivery. These attributes are particularly advantageous for addressing tumor heterogeneity and advancing personalized medicine approaches in oncology. Moreover, integrating advanced 3D tumor models with high‐throughput ssDNA probe selection presents a promising strategy for developing next‐generation cancer diagnostics and therapeutics. The selective targeting of prostate cancer cells within physiologically relevant 3D spheroids, combined with minimal off‐target effects and real‐time NIR fluorescence imaging, positions this platform as a powerful tool for non‐invasive cancer detection, monitoring, and therapy.

## 4 Methods

### 4.1 Prostate Cancer Cell Culture

The human prostate cancer cell line LNCaP was maintained in the RPMI1640 medium (Gibco), supplemented with 10% fetal bovine serum (FBS, Gibco) and 1% Pen‐Strep (Gibco). The cells seeded in 2D culture dishes were trypsinized (0.25% trypsin/EDTA; Sigma–Aldrich) and resuspended. The culture medium was refreshed roughly once every two or three days.

### 4.2 Bioink Formation

Porcine dermis was collected from a slaughterhouse (Majang‐dong), decellularized, and formulated into a bioink. The lyophilized tissues were dissolved in 0.5 M CH_3_COOH solution containing 10 mg of pepsin per 100 mg of dECM for seven days to produce the dECM bioink. After the complete dissolution of dECM, the pH of dECM bioink was adjusted to 7.4 with a 10 M NaOH solution. Before printing, LNCaP were encapsulated in 1% w/v dECM bioink with a density of 1 × 108 cells mL^−1^ and loaded in a sterile syringe connected to a 26G stainless steel microneedle. Each bioink group was placed in the PEVA (PolyScience) structure. A series of prostate spheroids was gradually printed within the dECM bath for ≈200 ms at a 3–6 kPa pressure on a predefined printing path. The printing pressure was changed while maintaining a constant time deposition (≈200 ms) to vary the diameter of the spheroids. The printing needle was withdrawn after printing the spheroids. The printed tissues were transferred into an incubator at 37°C for cross‐linking.

### 4.3 Characterization of Bioink

The rheological properties of dECM were measured using a rheometer (TA Instruments) with 25‐mm parallel plates. The storage and loss moduli were measured by performing the strain sweeps while increasing the strain from 0.01% to 100%. The shear recovery behavior of dECM samples with various concentrations was measured by varying the shear strain at 1% and 100% at 15°C. The shear thinning effect was assessed by performing steady shear sweep analyses of dECM at 15°C. Scanning electron microscopy (SEM, Zeiss Gemini 500) was performed after thermal cross‐linking for 40 min. The dECM bioink was freeze‐dried at −20°C for 24 h, and the cross‐sections were imaged at an accelerating voltage of 5 kV. The porosity and pore wall thickness of the bioink were analyzed using ImageJ. The swelling ratio was measured by comparing the weight of the dECM bioink after lyophilization and immersion in cell culture media for 24 h.

### 4.4 Cell Viability Test

The viability of LNCaP cells after encapsulation was evaluated using a Live/Dead Viability Kit (Invitrogen). All groups were incubated at 37°C for 40 min to induce thermal cross‐linking of the structures. After washing with PBS, the samples were transferred to a staining solution containing calcein AM and ethidium homodimer‐1 (EthD‐1) in PBS. The stained structures were observed under a fluorescence microscope (Axio Zoom, Zeiss). The cell viability was determined by calculating the ratio of viable cells to the total cell number.

### 4.5 Analysis of Immunofluorescence Staining

For the immunostaining experiments, the samples were fixed with 4% (w/v) paraformaldehyde solution. The cells were permeabilized with 0.1% Triton X‐100 solution (Biosesang) and treated with 1% (w/v) bovine serum albumin (Life Science) solution to reduce the non‐specific background. The samples were incubated with the primary antibodies overnight at 4°C. After washing gently with 1 × PBS, the secondary antibodies, Alexa Fluor 488 and 594, were added to the sample and incubated for 2 h at room temperature. All samples were counterstained with DAPI (Thermo Fisher Scientific) for 1 h. The stained samples were imaged by confocal microscopy (LSM 900, Zeiss).

### 4.6 mRNA Expression Analysis

mRNA expression was analyzed by extracting the RNA of prostate cancer cells in dECM bioink using the phenol/chloroform method with TRIzol reagent (Ambion) and chloroform (Sigma–Aldrich). The extracted mRNA was collected and precipitated using isopropyl alcohol (IPA) and glycogen (Roche). The precipitated RNA pellets were washed with 75% ethanol in diethylpyrocarbonate‐treated water (Biosesang). After dehydrating the RNA pellets, the RNA samples were dissolved in diethylpyrocarbonate‐treated water. The quantity and purity of the extracted total RNA were measured using a nanodrop spectrophotometer (Thermo). The purified total RNA was reverse transcribed to complementary DNA using an iScript cDNA Synthesis Kit (Bio‐Rad). Gene expression was quantified with SYBRgreen using the LightCycler 480 System (Roche). The primers were designed by referring to published gene sequences (NCBI)

### 4.7 Selection Protocol for ssDNA‐SWCNT Probes

The Initial Random ssDNA Library, with the Sequence 5`‐AGCGTCGAATACCACTAC‐N30‐ACCACGAGCTCCATTA G‐3` (Integrated DNA Technologies), included 18‐mer primers for PCR amplification. In the first round, 1 mg of SWCNTs and 1 mg of the ssDNA library were mixed in PBS and bath‐sonicated for 2 min. Subsequently, tip sonication (VCX130, 3 mm probe, Sonics) was performed for 30 min at 4 W (50% amplitude) in an ice bath. After sonication, the SWCNT dispersion was centrifuged at 21 000 × g for 60 min to remove the undispersed SWCNTs. The supernatant containing the ssDNA‐SWCNT complexes was then spin‐filtered five times at 6000 rpm using a 100‐kDa molecular weight cutoff filter (Amicon ultra‐0.5, Millipore) with DNase‐free water to eliminate the unbound ssDNA. The ssDNA‐SWCNT was collected and diluted with PBS. The concentration was calculated by measuring its absorbance at 632 nm using the SWCNT extinction coefficient (0.036 mg·L^−1^ cm^−1^) [56].

For positive cell screening, 10 mg/L of ssDNA‐SWCNTs library was incubated with 4 × 10^6^ of LNCaP cells as the target cell, washed three times with 1 × PBS buffer for 1 h at 4°C on a shaker. After incubation, ssDNA‐SWCNT‐bounded LNCaP cells were washed twice with 3 mL of 1 × PBS to remove unbound ssDNA‐SWCNT, and targeted cells were collected using a scraper and dispersed in 500 µL of D.I. water. The ssDNA bound to the LNCaP cell surface was detached by heating the purified ssDNA‐SWCNTs at 95°C for 1 h in a dry bath. The desorbed ssDNA was collected by centrifugation at 21 000 × g for 10 min to precipitate any aggregated SWCNTs and cells. The supernatant was subjected to PCR amplification using a FAM‐labeled forward primer (FAM‐AGCGTCGAATACCACTAC) and a biotinylated reverse primer (biotin‐CTAATGAGACTCGTGGTC). The reaction mixture contained 5 U of Top DNA polymerase (Bioneer), 1× reaction buffer, 1 µM forward and reverse primers, 1 mM dNTPs, and 200 ng/mL of the ssDNA library template, prepared in 100‐µL volumes in 96‐well plates. A negative control without the ssDNA library template was also included. The PCR cycling conditions consisted of an initial denaturation step at 95°C for 60 s, followed by N cycles (10–20) of denaturation at 95°C for 20 s, annealing at 60°C for 30 s, and extension at 72°C for 40 s, with a final extension at 74°C for 60 s. The number of cycles, N, was determined based on preliminary PCR runs that produced the maximum ssDNA yield with minimal byproducts. After PCR, 100 µL of the product was purified using a GeneJET PCR Purification Kit (Thermo Fisher Scientific) for sequencing library preparation.

The PCR products were verified using electrophoresis in a 4% agarose gel (Bioneer), stained with SYBR Gold, and running in 1 × tris‐borate‐EDTA buffer for 20 min at 110 V. The DNA bands were visualized under blue LED light. The ssDNA from the PCR product was isolated by placing 2 mL of streptavidin‐coated beads (Pierce High‐Capacity Streptavidin Agarose, Thermo Scientific) in a sintered glass Buchner funnel (pore size <10 µm) and washed with 10 mL of DNase‐free water. The PCR product was incubated with the beads for 30 min to bind dsDNA, and the beads were washed twice with 20 mL of water. The FAM‐labeled ssDNAs were eluted by incubating with 8 mL of 0.2 M NaOH for 10 min and filtering. The eluted ssDNAs were desalted using a NAP‐10 desalting column (Glen Research) and concentrated using a freeze dryer. Finally, the ssDNA concentration was measured by absorbance at 260 nm, yielding ssDNA for the next selection round. In Round 4, 4 × 10^6^ HDF cells were incubated with 10 mg/L of the ssDNA‐SWCNTs library as the control cells for negative cell screening and washed three times with 1 × PBS buffer. The resulting mixture was incubated on a shaker for 1 h at 4°C, and the supernatant of DNA‐SWCNTs that was unbound on the HDF cells was collected. The subsequent positive selection was performed by incubating the target cells with the collected solution on a shaker at 4°C for 1 h. The experiments were conducted in the same order as in the previous round. The next round library was obtained, and the negative and positive rounds were repeated until round 7. From rounds 4–7, selection for 3D spheroids was also performed separately, and negative selection was not conducted. The experiment was performed on 180–360 3D spheroids printed with a diameter of 900 µm. For the elution of ssDNA from spheroid‐bound ssDNA‐SWCNTs, the spheroids were incubated in a dry bath at 95°C for 1 h, and the supernatant was collected using a 100‐kDa molecular weight cutoff filter (Amicon ultra‐0.5–100, Millipore) to remove the dECM component and passed through a 10‐kDa molecular weight cutoff filter (Amicon ultra‐0.5–10, Millipore) again to concentrate the mixture. Subsequent experiments were conducted using the same procedure.

### 4.8 DNA Sequencing and Analysis

Sequencing libraries obtained through PCR were prepared using the TruSeq Nano DNA kit and sequenced using a paired‐end 151 bp approach on the Illumina NovaSeq 6000 platform at ROKIT Genomics. The TruSeq universal adapter sequence used was AGATCGGAAGAGC. The libraries generated 6–11 million raw sequencing reads from rounds 6 and 7 in the 2D culture and 3D spheroid. The sequences were preprocessed to filter out the unique random 30‐mer regions fixed by two primers and determined the sequence counts. All experiments were performed using ssDNA sequences without the PCR primer regions. For the selection of representative probes, the entire pool of ssDNA sequences was clustered using the AptaCluster open‐source tool because the top sequences with high read numbers in the library were composed of highly similar sequences. The diversity of the test sequences was increased by selecting a representative top 10 group by clustering. The sequences with high read numbers within each group were selected as representative sequences.

### 4.9 Synthesis of ssDNA‐SWCNT Probes and Improvement of the Probes

In this procedure, 1 mg of HiPCo SWCNTs and 100 nmol of 1 mM ssDNA were combined in 0.9 mL of PBS. The mixture was initially bath‐sonicated for 2 min, followed by tip sonication for 20 min at 4 W power in an ice bath. After sonication, the ssDNA‐SWCNT solution was centrifuged at 21 000 × g for 1 h to precipitate the undispersed SWCNTs, and the supernatant containing solubilized ssDNA‐SWCNTs was collected. The supernatant was then spin‐filtered using 100 kDa MWCO centrifuge filters at 6500 rpm for 5 min with DNase‐free water. This spin filtration step was repeated three times to remove the unbound ssDNA. Finally, the ssDNA‐SWCNT constructs were collected and diluted with PBS. The concentration was determined by measuring the absorbance at 632 nm using the SWCNT extinction coefficient (0.036 mg·L^−1^·cm^−1^).

The target efficiency was enhanced by adjusting the SWCNT/DNA mass ratio from 1:1 to 1:3 with a fixed SWCNT concentration of 20 mg/L. The fabricated probes were incubated with LNCaP cells in 2D cultures for various durations, ranging from 1 to 20 s at RT. 1, 2‐dipalmitoyl‐sn‐glycero‐3‐phosphoethanolamine‐N‐[methoxy (polyethylene glycol)‐2000] (880160P, Avanti Polar Lipids, Inc.) and 1, 2‐dipalmitoyl‐sn‐glycero‐3‐phosphoethanolamine‐N‐[methoxy(polyethylene glycol)‐5000] (880200P, Avanti Polar Lipids, Inc.) were introduced as phospholipid‐PEG to minimize the non‐specific binding. The dispersed ssDNA‐SWCNTs were functionalized with phospholipid‐PEG via bath sonication for 15 min through noncovalent interactions to minimize adsorption and accumulation on the HDF surface as a negative control and dECM. A SWCNT/phospholipid‐PEG mass ratio of 1:1 and 1:4 was used, fixing the SWCNT concentration to 20 mg/L. The functionalized probes were incubated with LNCaP and HDF cells for 20 s at room temperature. In addition, this study evaluated the effects of increasing the PEG length (PEG 2000 vs. PEG 5000) on reducing non‐specific binding. Subsequent experiments followed the same protocol.

### 4.10 Evaluation of Probes via NIR Fluorescence in 2D Cell Culture and 3D Spheroid Model

For NIR‐II imaging, an epifluorescence inverted microscope system based on an Olympus IX73, equipped with two cameras, was used to image NIR‐II and visible light simultaneously. Visible light was excited using an LED light source and imaged with a sCMOS camera (CS2100M‐USB, Thorlabs). NIR‐II fluorescence was excited using a 721‐nm laser and imaged with an InGaAs camera (Ninox 640, Raptor Photonics). All cell experiments involving NIR‐II imaging were performed with a 500 ms exposure time. For 2D cell cultures, 100 µL of 20 mg/L phospholipid‐PEG/ssDNA‐SWCNT probes in 1 × PBS were added after the cells were washed three times with 1 × PBS. The probes were incubated for 1 min at RT and adsorbed onto the surface of the LNCaP cells. The unbound probes were removed by washing the cells three times with 1 × PBS before mounting the plate for NIR‐II fluorescence imaging.

In the 3D spheroid model, 100 µL of 50 mg/L phospholipid‐PEG/ssDNA‐SWCNT probes in 1 ×PBS were incubated for 10 min at RT on LNCaP spheroids that had been washed three times with 1 × PBS. After the nanoprobes were adsorbed onto the spheroid surfaces, the unbound probes were washed off, and NIR‐II imaging was performed to compare the difference between the signals from the nanoprobes.

The fluorescence intensity of the probes in 2D cell cultures was quantified by analyzing the images from bright field microscopy, F‐actin staining, FA‐labeled probe staining, or NIR‐II imaging using ImageJ. The Interactive H‐Watershed plugin was used to segment the cell boundaries and mask the intracellular regions by adjusting the seed dynamics and intensity thresholds in bright‐field images or F‐actin staining images, highlighting the cytoskeleton and overall cell structure. The resulting mask was overlaid on the NIR‐II or FAM‐labeled probe images to extract the fluorescence intensity per unit area within each cell (ROI). The average fluorescence intensity of approximately 100 cells within the FOV was calculated, and the results were compared across different probes.

For the LNCaP 3D spheroid models, images obtained from bright‐field, FAM‐labeled probe staining, or NIR‐II imaging were also analyzed using ImageJ. Because the spheroids displayed an approximately circular shape in the images, a manual ROI was drawn around each spheroid, and the fluorescence intensity within this area was measured. For each spheroid, the fluorescence intensity was quantified across three distinct FOVs, with one spheroid per FOV analyzed. The average fluorescence intensity from these FOVs was calculated, enabling a comparative analysis of the fluorescence between the different probes.

### 4.11 Cell‐Based Binding Affinity Assay

The binding affinity of two probe candidates was assessed by NIR‐II fluorescence imaging. Before performing the cell‐based binding affinity assay, different incubation times were evaluated to determine when the binding reaction reached equilibrium, which occurs when the fraction of the complex formed between two molecules remains constant over time. Binding and other kinetic processes typically followed exponential curves (ref). The equilibration time was estimated by incubating 4 × 10^6^ target LNCaP cells with the probes for varying durations (1, 5, 10, 20, 30, and 60 min). The binding reached saturation after 20 min, indicating that equilibrium had been achieved.

The NIR‐II fluorescence of bound probes was measured under the equilibrium conditions to determine the equilibrium dissociation constant (K_d_) for the interaction between the probes and the proteins expressed on the surface of the LNCaP cells. The probes were incubated with the cells at concentrations ranging from 100 pM to 200 nM. After 20 min, the cells were washed with 1 × PBS buffer to remove the unbound probes. The membrane‐bound probes were quantified via NIR‐II fluorescence, allowing for an evaluation of high‐affinity binding with specificity. The K_d_ is equal to the probe concentration when 50% of the membrane receptors are bound by probes. This value can be extrapolated from the fitted sigmoidal curve. After fluorescence normalization, the data were fitted using the Hill equation to obtain the K_d_ value.

### 4.12 Endocytosis Study for Probes in 2D Cultures

An endocytosis inhibitor study was conducted to investigate the cellular uptake pathway of probes. One day before the experiment, the LNCaP cells were seeded in a 96‐well plate at a density of ∼1 × 10^4^ cells/well. The cells were grown to 80%–90% confluency. After washing three times with 1×PBS buffer, the cells were preincubated with inhibitors specific to clathrin‐mediated endocytosis, phagocytosis, and macropinocytosis. Two inhibitors were tested for clathrin‐mediated endocytosis: hypertonic sucrose and potassium ion depletion. In the hypertonic sucrose method, the cells were treated with 0.45 M sucrose in buffer (50 mM HEPES, 100 mM NaCl, 1 mM CaC1_2_, 10 mM KCl in pH 7.4) at 37°C for 10 min, followed by washing with the same buffer (without sucrose) at 4°C. In the potassium ion depletion method, the cells were washed twice with potassium depletion buffer (140 mM NaCl, 20 mM HEPES, 1 mM CaCl_2_, 1 mM MgCl_2_, and 1 mg/mL D‐glucose in pH 7.4) and incubated with a hypertonic buffer (a 1:1 mixture of potassium depletion buffer and H_2_O) for 15 min at 37°C, followed by washing three times with potassium depletion buffer. Phagocytosis and micropinocytosis were evaluated by treating the cells with 5 µM cytochalasin D in DMEM (BSA, 20 mM HEPE) and incubating at 37°C for 30 min.

After preincubation with the inhibitors, the cells were incubated with 100 µL of 20 mg/L probe for 10 min at RT. The unbound probes were removed. The cells were washed with PBS and fixed with 4% paraformaldehyde at 37°C for 15 min. The untreated cells were used as a negative control, while the cells treated only with the probes without inhibitors were used as the positive control. Imaging was performed to assess the changes in the NIR‐II fluorescence intensity, and the fluorescence intensity was quantified using ImageJ. The results were compared with the positive control to evaluate the extent of endocytic uptake of the probes.

### 4.13 Fluorescence‐Activated Cell Sorting (FACS)

LNCaP spheroids were cultured for four days and incubated with either FAM‐conjugated SWCNT control or experimental ssDNA–SWCNT probes at 20 mg/L for 10 min at room temperature. FAM‐(ACG)_6_ ssDNA was used to detect the specific cell populations via flow cytometry. The (ACG)_6_ sequence was chosen for its shorter length, and the CG composition was adjusted to ensure moderate binding affinity without exceeding the binding affinity of the target sequence on the SWCNT surface. After incubation, the samples were washed three times with PBS. Each wash was performed for 3 min at room temperature to ensure the sufficient removal of unbound probes.

The spheroids were then enzymatically dissociated using collagenase (Sigma–Aldrich) for 40 min at 37°C to release the single cells from the dECM matrix. The resulting suspensions were filtered through a 40 µm cell strainer to ensure single‐cell separation and transferred to FACS tubes for analysis.

Flow cytometry was performed using a BD FACSCanto II (BD Biosciences) equipped with a 488 nm laser, and a 70 µm nozzle was fixed. The samples were acquired using BD FACSDiva 8.0.1 software under standard instrument settings. FSC‐A vs. SSC‐A gating was used to exclude the debris, and FSC‐H vs. FSC‐A gating was applied to isolate the singlet cell populations. Probe‐positive and probe‐negative populations were distinguished based on FITC‐A fluorescence intensity. Compensation was performed automatically. At least 10 000 events per sample were collected and analyzed using FlowJo software (version 9.2).

### 4.14 Drug Delivery of ssDNA‐SWCNT Probes Conjugated With Doxorubicin

The SWCNTs were synthesized with the target ssDNA and NH_2_‐(ACG)_6_ ssDNA. The DOX was attached to the SWNTs through a hydrazone bond according to the method described previously [71]. The SWCNT‐NH_2_ (250 mg/L, 400 µL) was suspended with HBA (3.0 mg) in a pH 7.4 phosphate‐buffered saline (PBS) solution (500 µL), EDC⋅ HCl (4.8 mg), and NHS (2.0 mg) were then added. The mixture was allowed to react at room temperature for 24 h, after which the conjugate was dialyzed against H_2_O to remove the unreacted reagent to obtain the SWNT‐HBA complex. Finally, the solution was freeze‐dried to obtain the hydrazine‐modified SWNTs as a fine powder. DOX can be bound to SWNTs by a reaction of SWNT‐HBA with DOX⋅ HCl in dimethyl sulfoxide (DMSO) in the dark. Freeze‐dried hydrazine‐modified SWNTs (1 mg) were dissolved in 100 µL anhydrous DMSO, and an excess of DOX⋅ HCl (0.1 mg) was added. The mixture solution was stirred at room temperature in the dark for 24 h. After the reaction, the solution was centrifuged with dialysis tubing (MWCO of 100 kDa) at 6500 rpm to remove the excess unbounded DOX molecules. The amount of DOX loaded onto the SWNTs was measured using the absorbance peak at 490 nm (characteristic of DOX, after subtracting the absorbance of SWNTs at that wavelength) based on a standard curve of DOX. The rate of DOX released from the SWNTs was measured as a function of time during incubation in 100 mM PBS at pH 7.0 and pH 5.0. The samples were then incubated at room temperature. The nanotubes were centrifuged with dialysis tubing (MWCO of 100 kDa) to collect the released DOX. The amount of released DOX was analyzed by UV–vis spectrophotometry at 490 nm.

### 4.15 Statistical Analysis

Prism version 8 (GraphPad) software was used for statistical analysis. Statistical comparisons between two experimental groups were performed using an unpaired two‐tailed *t*‐test, and the groups were compared using one‐way analysis of variation (ANOVA) with multiple comparison tests. The *p*‐values are represented as asterisks on graphs (^*^ *p* <0.05; ^**^ *p* <0.01; ^***^ *p* <0.001; ^****^ *p* < 0.0001). All experimental values represent a minimum of three individual experiments.

## Author Contributions

S.J., B.S.K., S.K., and S.O. conceptualized the study. D.L., S.L., Y.J., J.L., and M.C. conducted the experiments with support from S.J. and B.S.K. The SELEC experiment was performed by Y.J. Probe optimization, evaluation, cellular uptake, and drug delivery studies were carried out by D.L. S.L. conducted 3D bioprinting, spheroid characterization, FACS study, and confocal imaging. J.L. and M.C. provided technical aids for the bioprinting experiments. D.L. and S.L. designed the figures and wrote the manuscript. S.J. and B.S.K. edited the manuscript. All authors approved the manuscript.

## Conflicts of Interest

The authors declare no conflicts of interest.

## Supporting information




**Supporting File**: advs73899‐sup‐0001‐SuppMat.docx.

## Data Availability

The data that support the findings of this study are available in the supplementary material of this article.
