# Supplementary material for: Microenvironment‐Guided Evolution of ssDNA‐SWCNT Probes for Selective Recognition of Aggressive Prostate Cancer Phenotypes
Source: Adv Sci (Weinh). 2026 Jan 21;13(17):e18582. doi: 10.1002/advs.202518582 (PMC13042387; doi:10.1002/advs.202518582)
Supplement: Supplementary file 1 — Supporting File: advs73899‐sup‐0001‐SuppMat.docx. [file ADVS-13-e18582-s001.docx]

**Supporting Information**

**Microenvironment-guided Evolution of ssDNA-SWCNT Probes for Selective Recognition of Aggressive Prostate Cancer Phenotypes**

Dakyeon Lee^1, 2†^, Seokhyeon Lee^1†^, Yunseo Jeong^1^, Jeongho Lee^1^, Minseo Choi^1^, SaeOck Oh^3^, Sungjee Kim^2*^, Byoung Soo Kim^1,4*^, Sanghwa Jeong^1*^

^1^School of Biomedical Convergence Engineering, Pusan National University, Yangsan, Republic of Korea.

^2^Department of Chemistry, Pohang University of Science and Technology, Pohang, Republic of Korea.

^3^Department of Anatomy, School of Medicine, Pusan National University, Yangsan 50612, Republic of Korea

^4^Research Institute for Convergence of Biomedical Science and Technology, Pusan National University Yangsan Hospital, Yangsan, Republic of Korea

Corresponding authors: sanghwa.jeong@pusan.ac.kr, bskim7@pusan.ac.kr, sungjee@postech.ac.kr


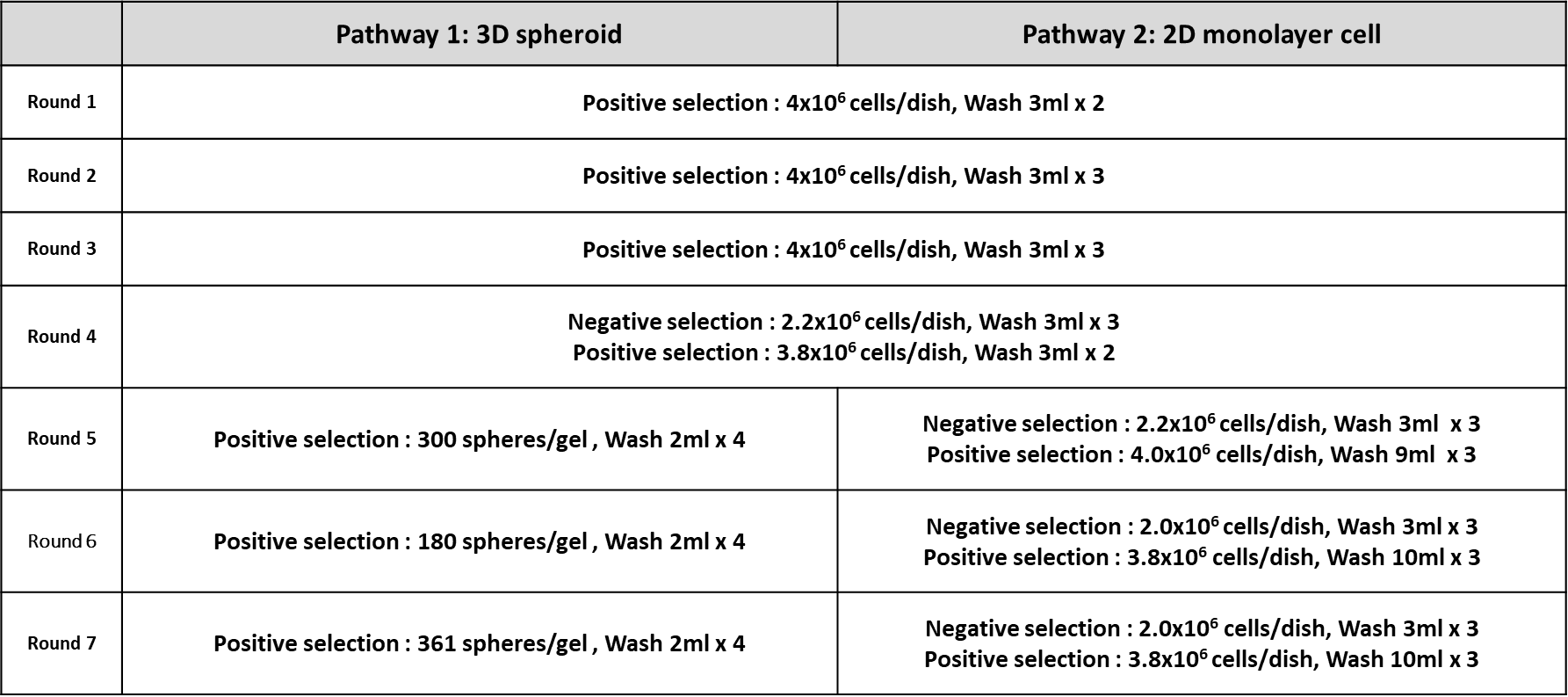


**Table S1. Summary of the SELEC screening conditions for prostate cancer-specific ssDNA-SWCNT probes under two selection pathways.** Detailed experimental parameters for Pathway 1 (3D spheroid model) and Pathway 2 (2D monolayer culture) across seven SELEC rounds. Both pathways initially followed positive selection using 2D-cultured LNCaP prostate cancer cells (Rounds 1–4). From Round 5, Pathway 1 transitioned to 3D spheroid selection to mimic a physiologically relevant tumor architecture, while Pathway 2 continued in the 2D format. Negative selection steps were introduced to reduce non-specific binding: incorporated once in Round 4 for Pathway 1 and Rounds 4–7 for Pathway 2.


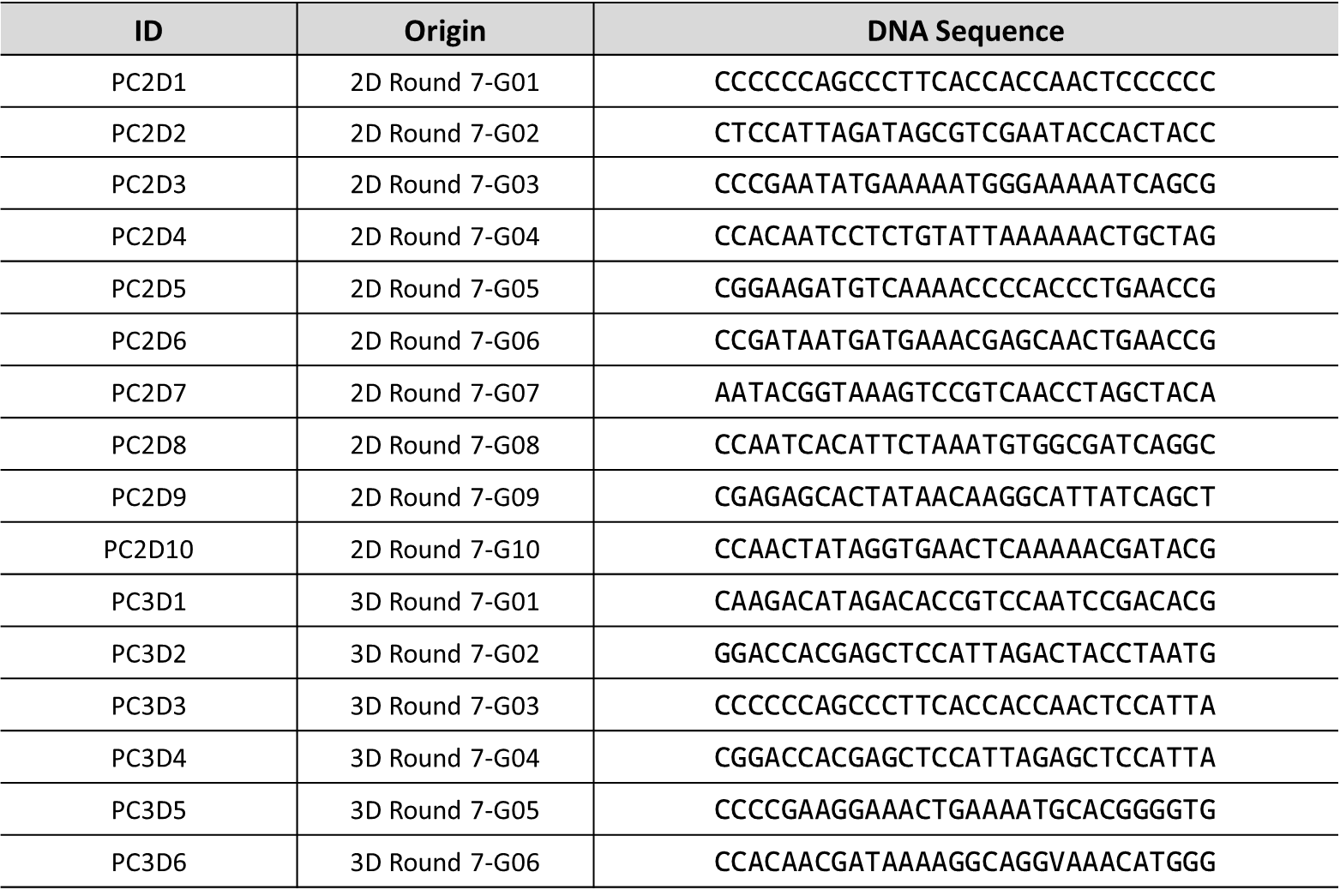


**Table S2.** **The table list of 16 probe candidates.** Candidate probe information includes 2D or 3D Cell-SELEC conditions, selection rounds, clustering group, and corresponding DNA sequence.


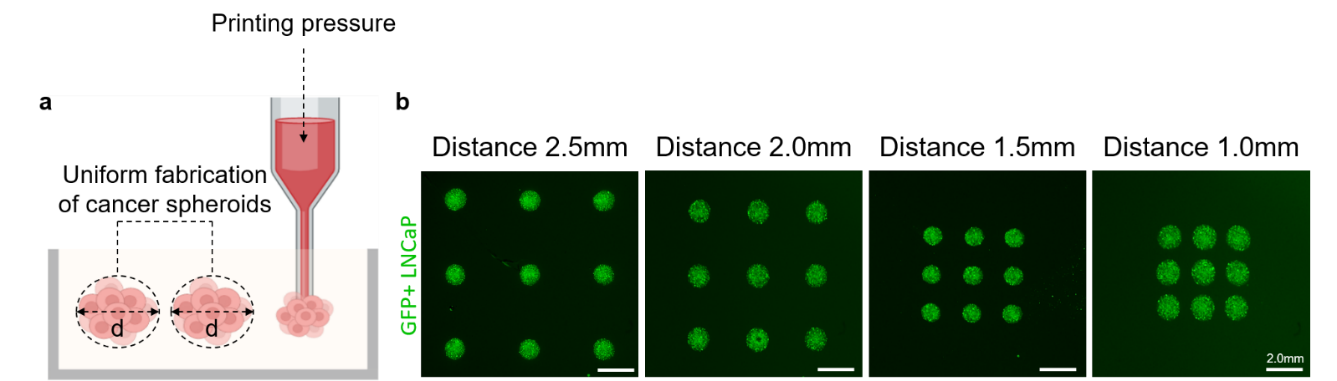


**Figure S1**. **Accurate spatial positioning achieved through G-code-based printing path design**. (a) Fluorescence microscopy images showing precise distance control based on G-code design using GFP-expressing LNCaP cells.


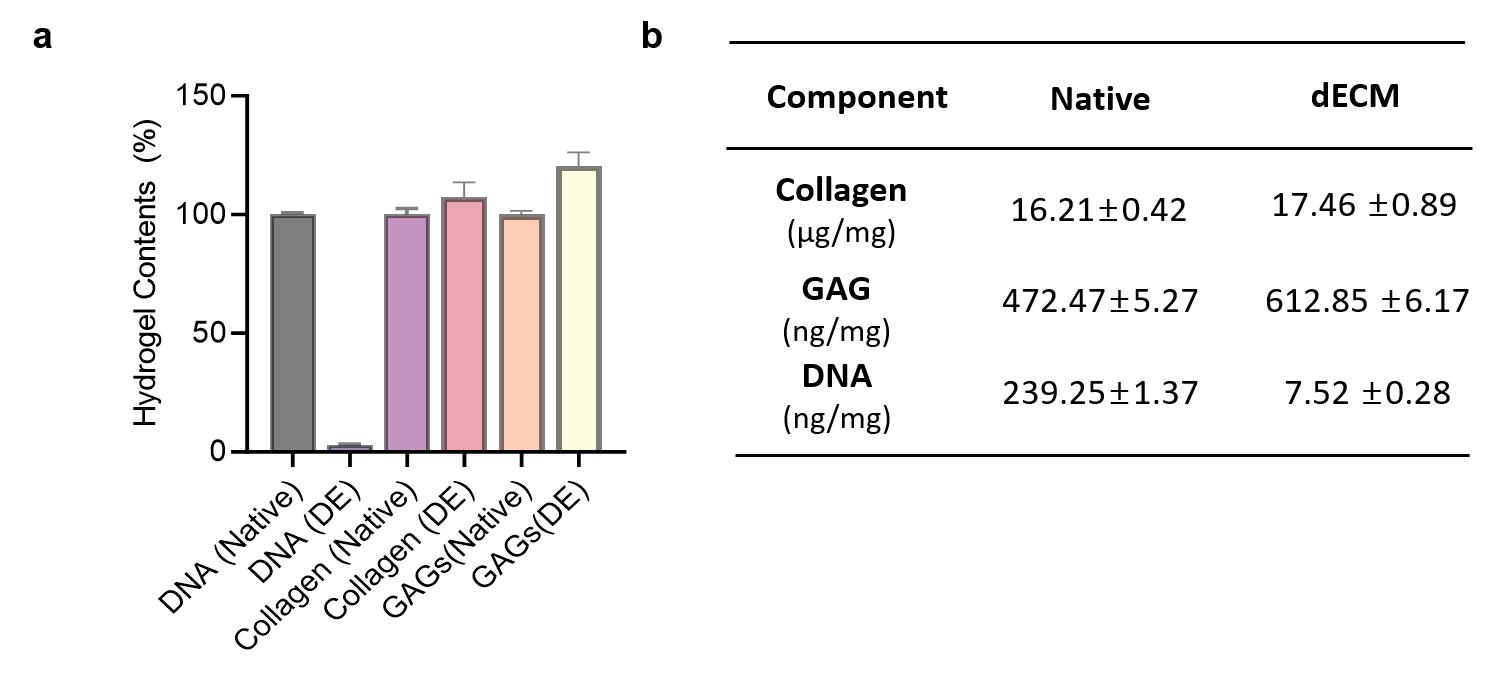


**Figure S2**. **Quantitative comparison of ECM components between decellularized and native tissue samples.** (a) Relative differences in collagen, glycosaminoglycans, and DNA content between native and dECM. (labeled as ‘DE’ in the graph) The decellularization process significantly reduces the DNA content while retaining substantial amounts of collagen and GAGs, essential for the bioactive properties of the dECM. (b) Quantitative comparison of collagen (µg/mg), glycosaminoglycans (GAGs, ng/mg), and DNA content (ng/mg) in dECM compared to native tissue samples. Data are presented as mean ± SD.


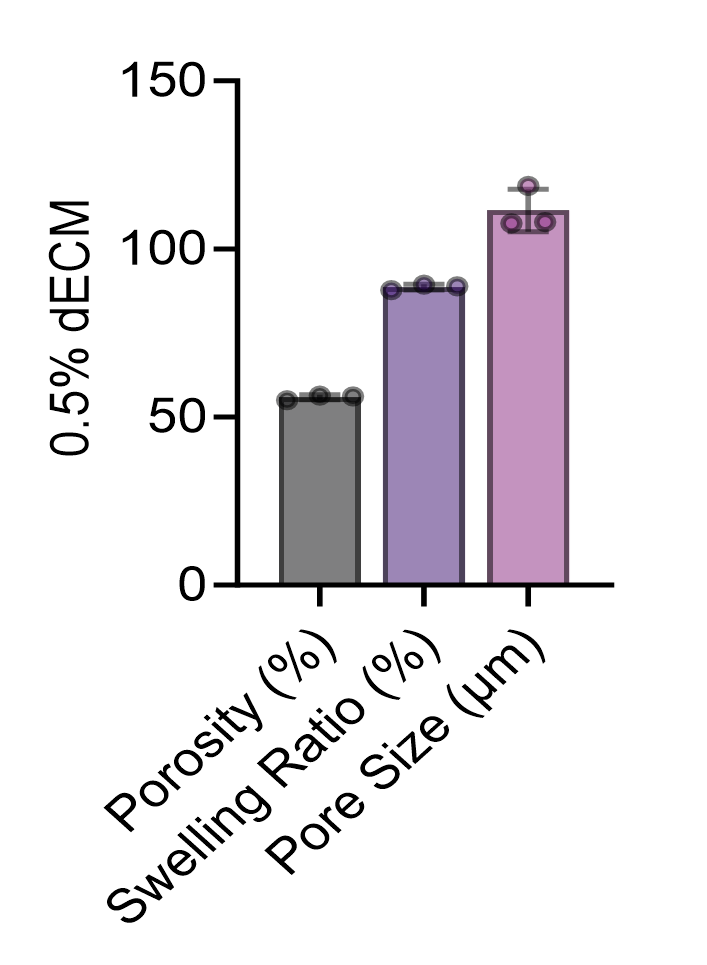


**Figure S3. Characterization of 0.5% dECM.** (a) Porosity and pore size of thermally cross-linked 0.5% dECM were measured using ImageJ. The swelling ratio was measured by comparing the weight of the bioink after lyophilization and immersion in PBS for 24 hours.


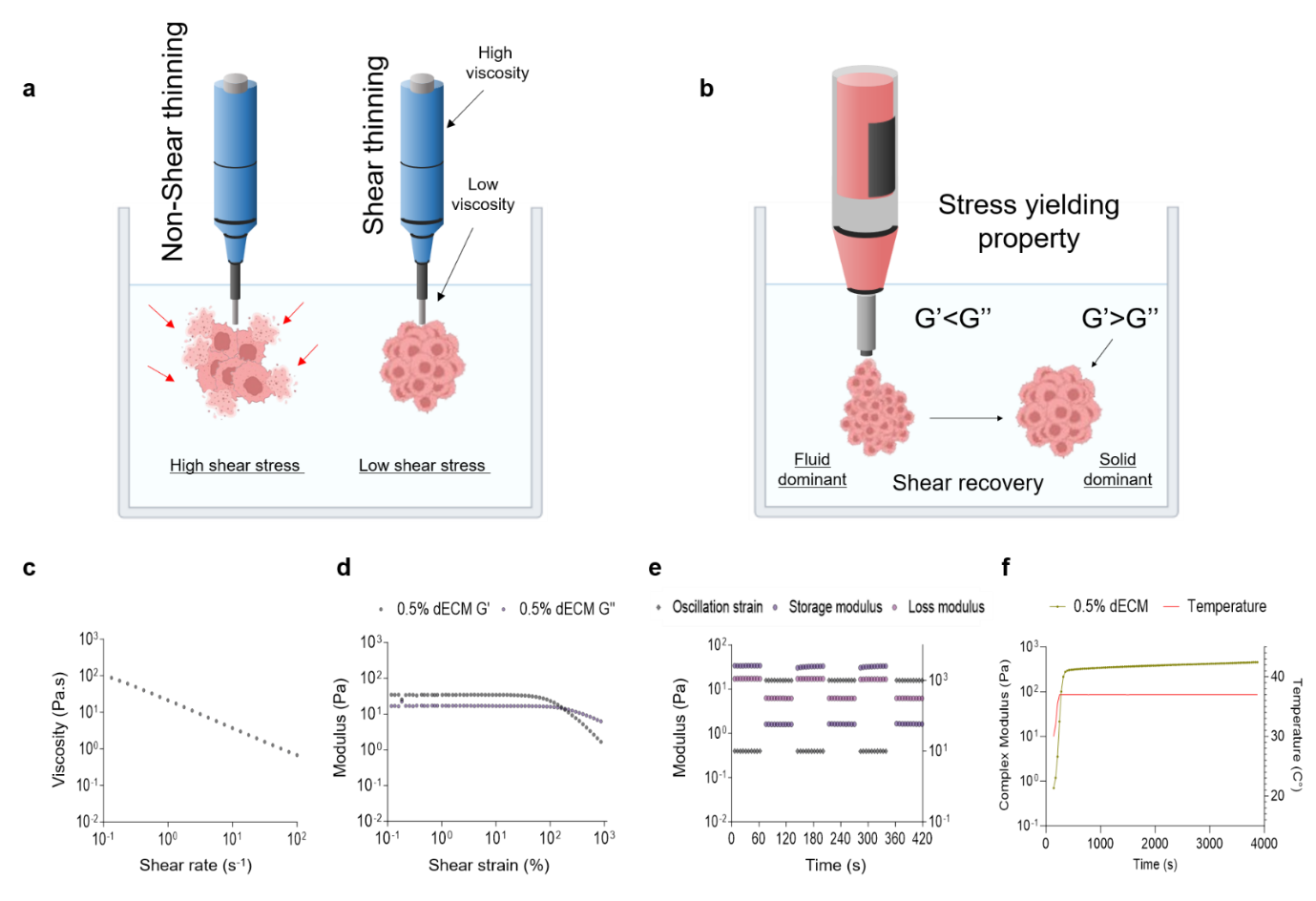


**Figure S4. Rheological evaluation of bioink.** (a) Schematic diagram of shear thinning in in-bath bioprinting. (b) Schematic diagram of Bingham plastic behavior and shear recovery in in-bath bioprinting (c) Rheology test results for shear thinning behavior of 0.5% dECM (d) Rheology test results for Bingham plastic behavior of 0.5% dECM (e) Rheology test results for shear recovery of 0.5% dECM (f) Evaluation of gelation kinetics of 0.5% dECM.

**
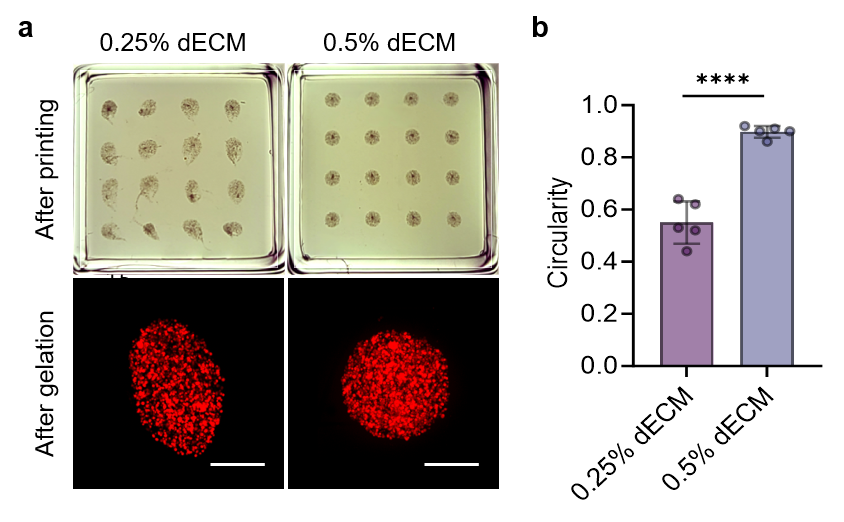

Figure S5. Printability assessment.** (a) Spheroid images printed using 0.25% and 0.5% bath concentrations, shown as optical microscopy images (top) and Cell Tracker Red–labeled LNCaP images (bottom). (b) Quantification of spheroid circularity obtained from spheroids printed in 0.25% and 0.5% baths.


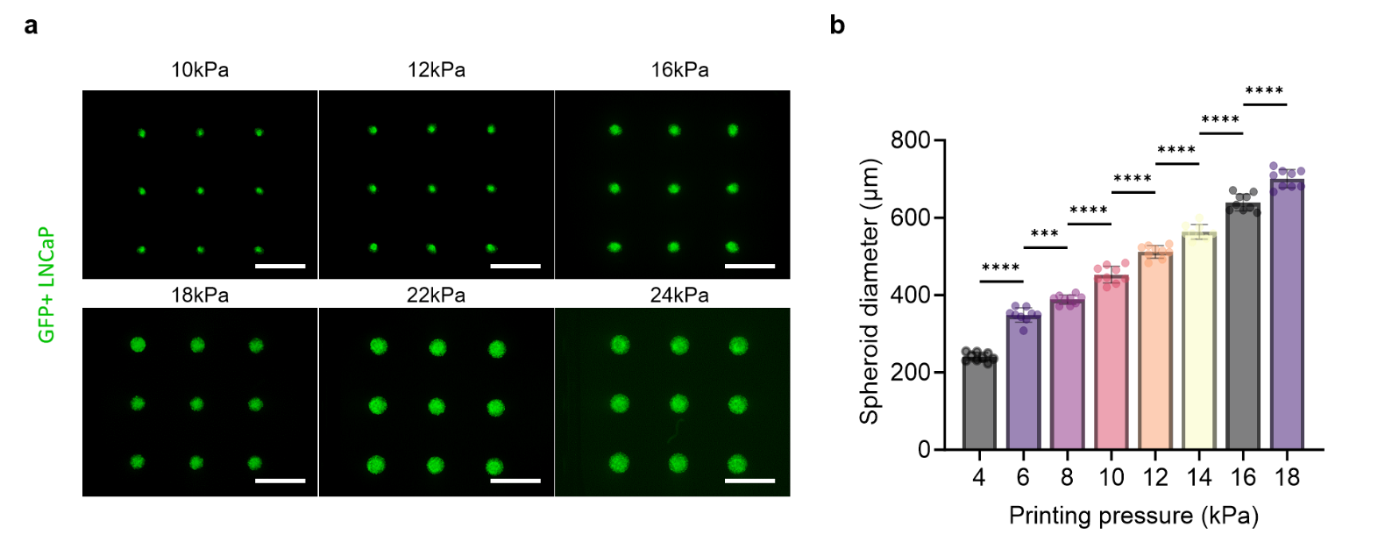
 **Figure S6. Establishment of the spheroid manufacturing process.** (a) Fluorescence microscopy images of spheroids printed under varying pneumatic printing pressures using GFP-expressing LNCaP cells.(b) Quantification of the spheroid size under different printing pressures.


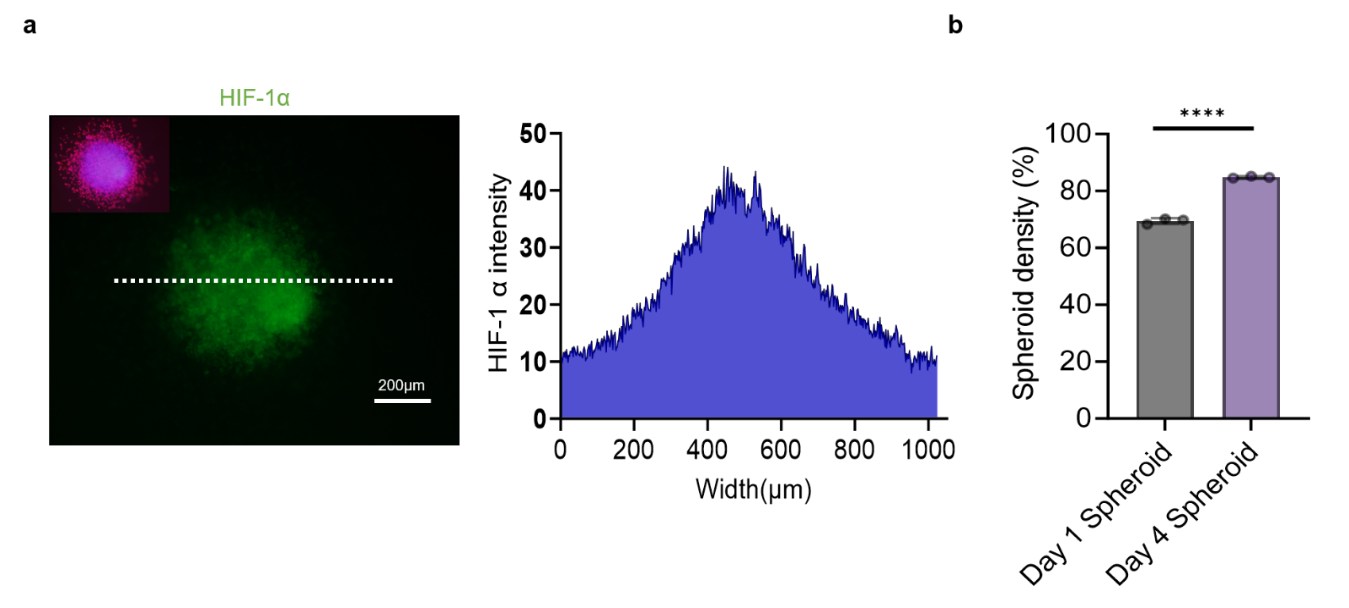
 **Figure S7. Hypoxia analysis of tumor spheroids** (a) HIF-1α intensity quantification graph by region. (b) Quantification graph of the spheroid density. The density was defined as the DAPI-positive area fraction within the total spheroid region.


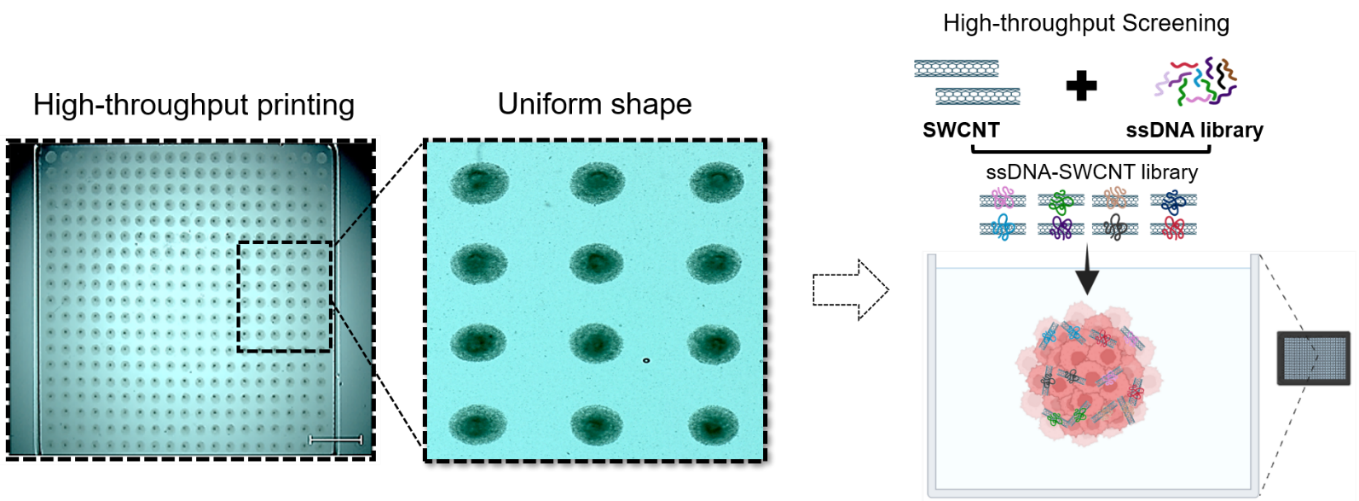


**Figure S8. High-throughput printing and high-throughput screening.** Optical microscopy image of a 20 × 20 spheroid array printed using the in-bath printing method. Schematic illustration depicting the workflow for high-throughput screening using the printed spheroid array.


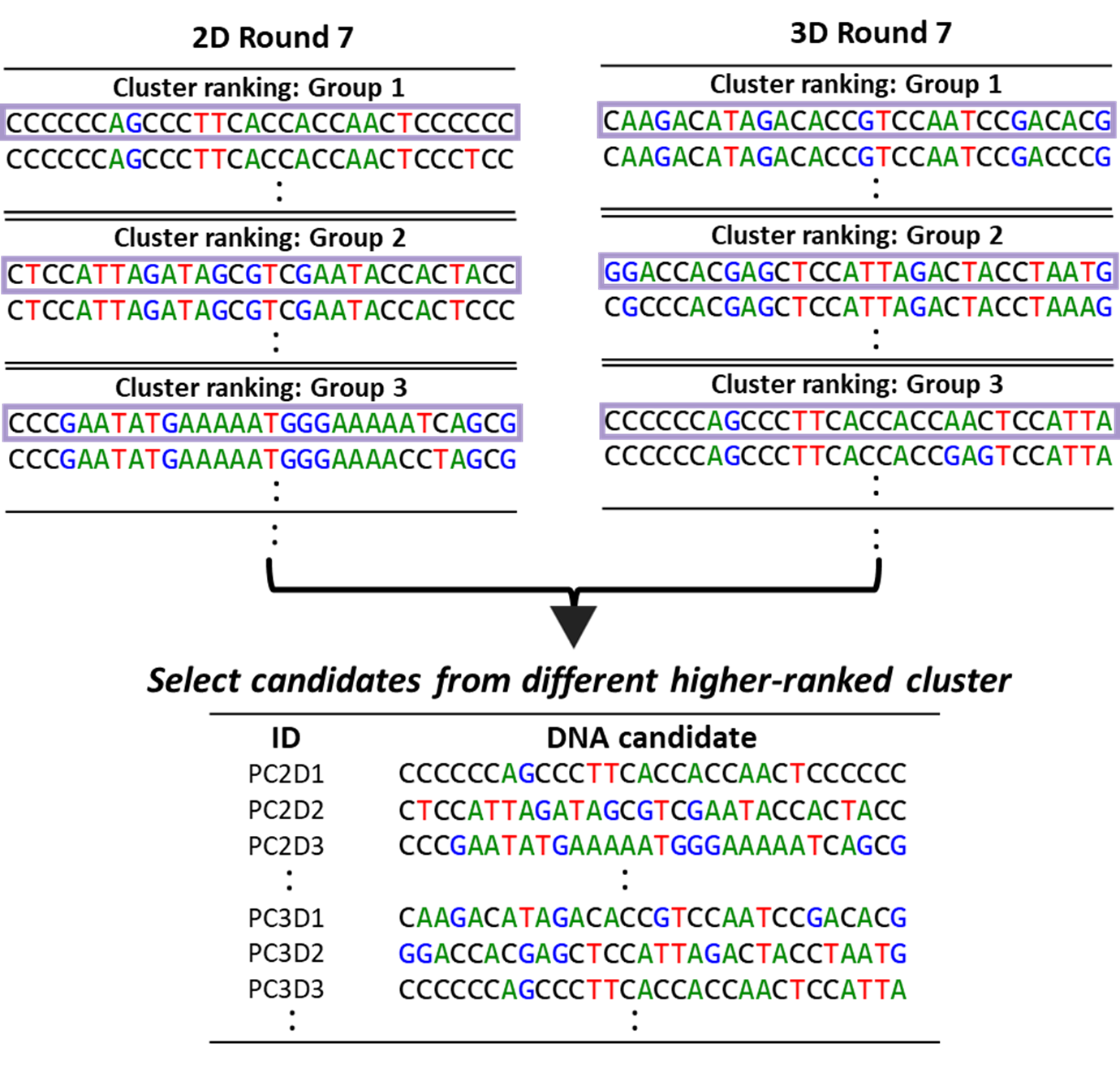


**Figure S9. Schematic diagram of the clustering process for identification of prostate cancer-specific ssDNA-SWCNT probe candidates.** High-throughput sequencing results from SELEC Round 7 (2D and 3D selections) were processed by clustering analysis using the AptaSUITE tool to enhance sequence diversity. For the 2D condition, the top 10 clusters were identified, and for the 3D condition, the top six clusters were selected while avoiding clusters with highly similar sequences. Within each cluster, the most abundant sequence was chosen as the representative candidate. These selected sequences were designated using the nomenclature PCND-M, where “PC” stands for prostate cancer, “N” denotes the culture condition (2D or 3D), and “M” indicates the rank order of the cluster. This approach resulted in 16 unique ssDNA sequences.


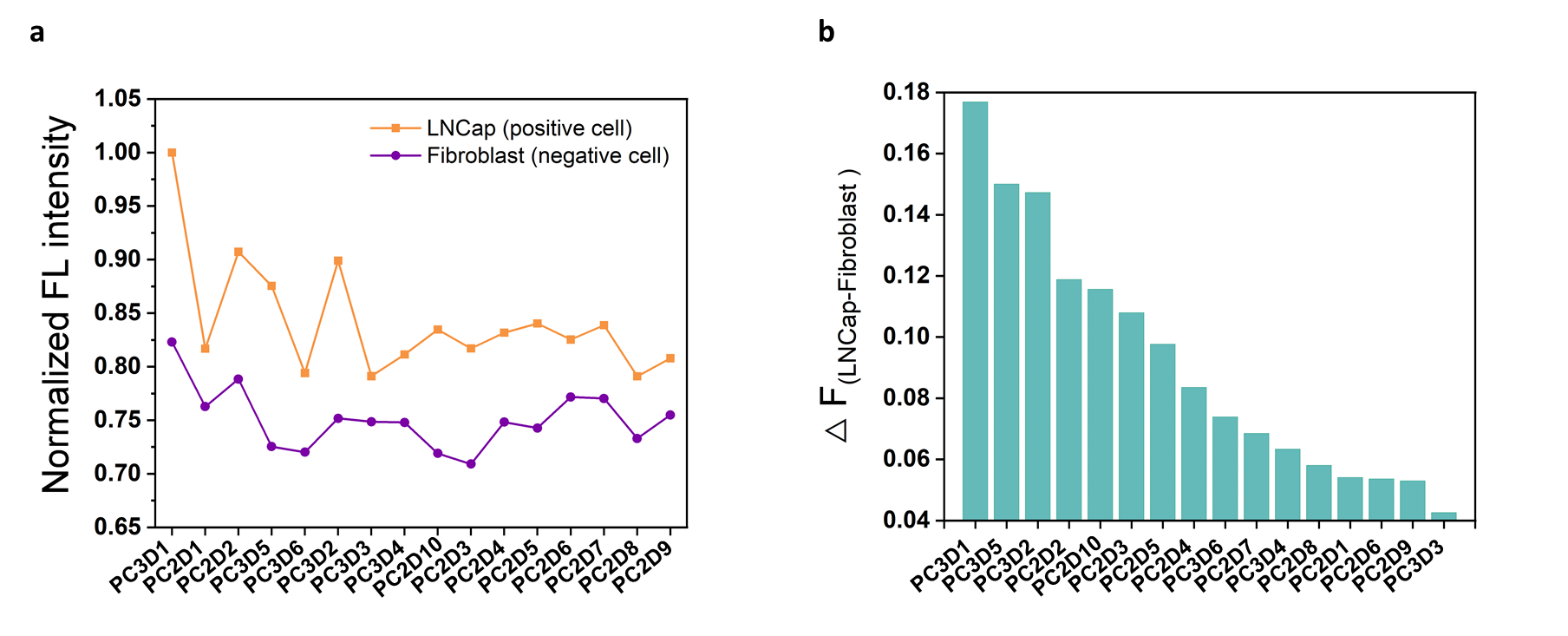


**Figure S10. Evaluation of probe performance in 2D cultures of LNCaP and fibroblast cells.** (a) The normalized fluorescence intensity of different proves in LNCaP as the target cell and fibroblast cells as the negative control. (b) Difference in fluorescence intensity between positive and cells, as shown in (a), with probes ranked in descending order based on the magnitude of fluorescence intensity difference.


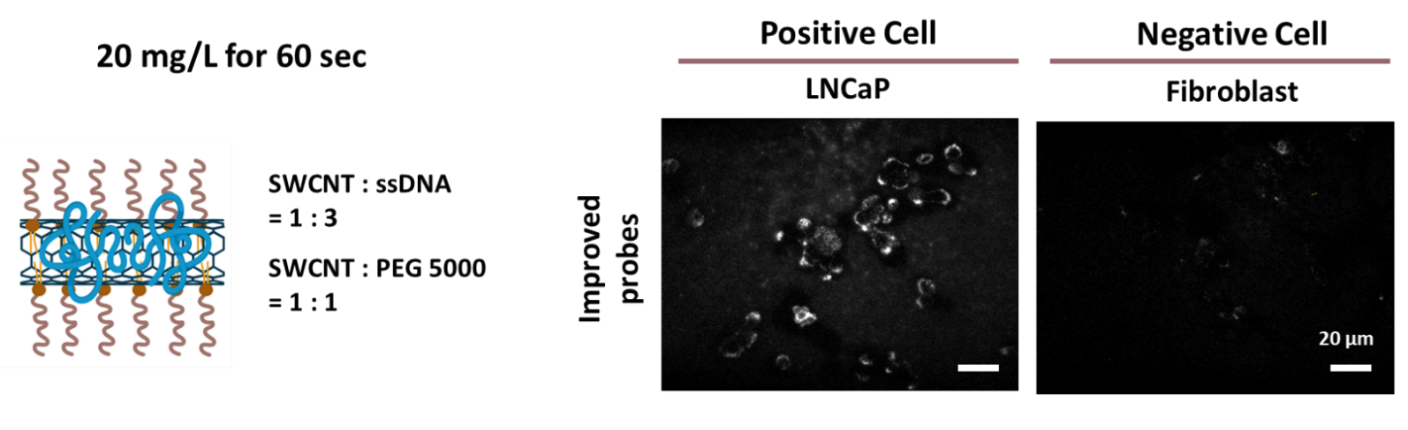


**Figure S11. Optimization of the probe treatment conditions in 2D cultures.** The illustration shows that the optimized probe achieved improved sensitivity and specificity by increasing the ssDNA/SWCNT mass ratio to 1:3 and incorporating phospholipid-PEG5000 with an SWCNT/PEG mass ratio of 1:1. The NIR-II images revealed a significant decrease in fluorescence intensity in fibroblast cells.


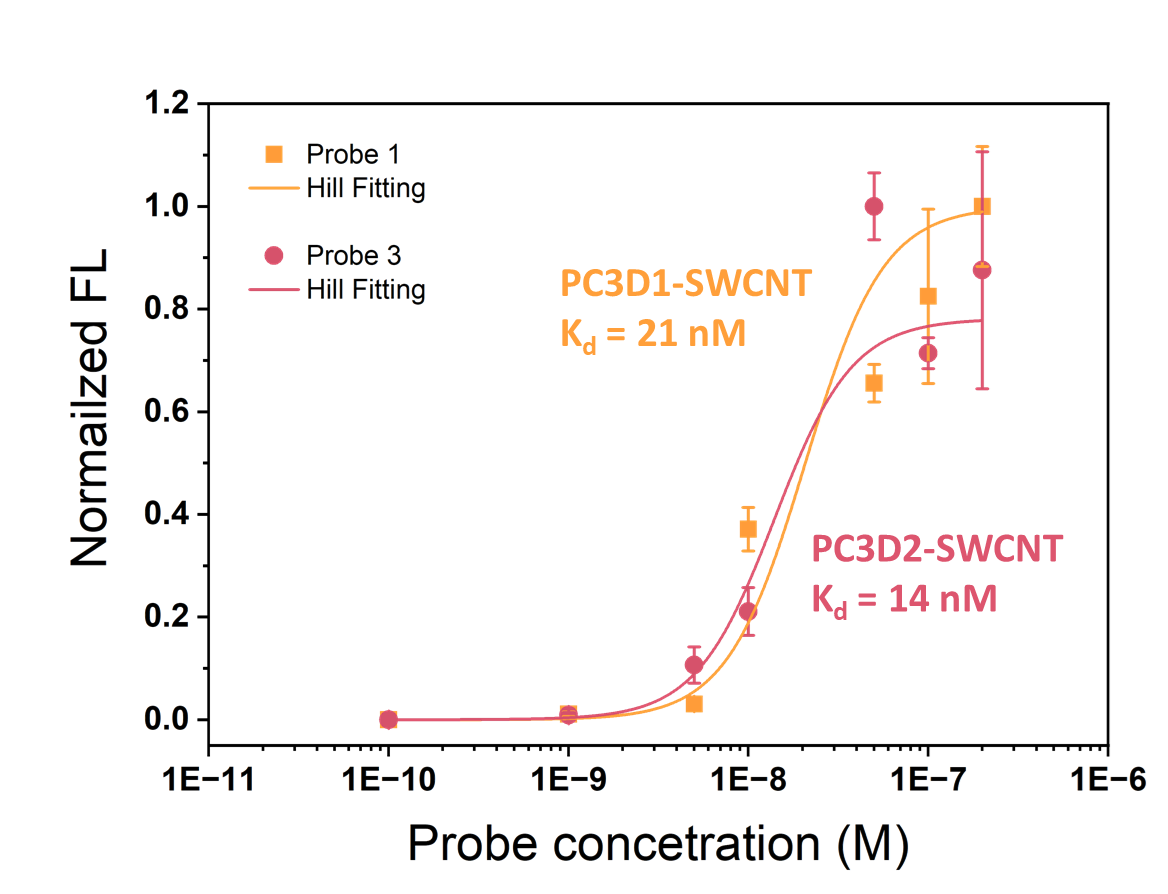


**Figure S12. Cell-based binding affinity assay of probes (PC3D1- and PC3D2-SWCNT).** The binding affinity of PC3D1- and PC3D2-SWCNT was assessed using NIR-II fluorescence imaging and calculated dissociation constant (K_d_) for the interaction between the probes and the proteins expressed on the surface of LNCaP cells. LNCaP cells were incubated with the probes for varying concentrations (100 pM, 1 nM, 5 nM, 10 nM, 50 nM,100 nM, and 500 nM). The K_d_ values of PC3D1-SWCNT and PC3D2-SWCNT are 21 nM and 14 nM, respectively.
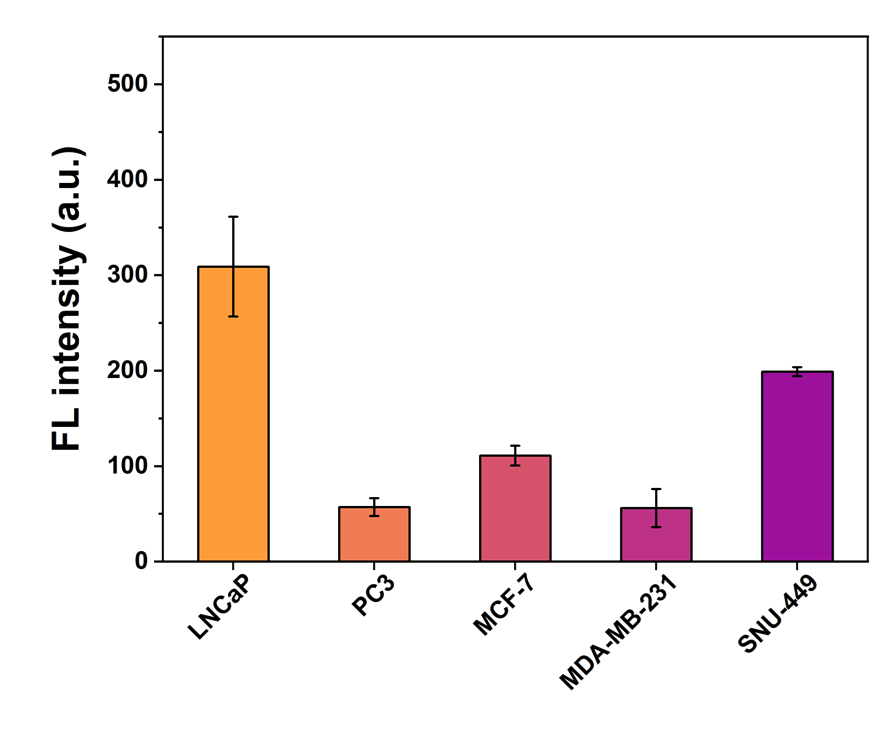


**Figure S13. Quantification of fluorescence intensity of PC2D2-SWCNT probes with other cell line spheroids.** The quantification of fluorescence intensity within spheroids in prostate (LNCaP, PC3), breast (MCF-7. MDA-MB-231), and liver (SNU-449) cancer cells. The fluorescence intensity was higher in LNCaP cells than PC3 cells, both of which are prostate cancer cell lines, indicating that PC2D2-SWCNT is specific to LNCaP cells.


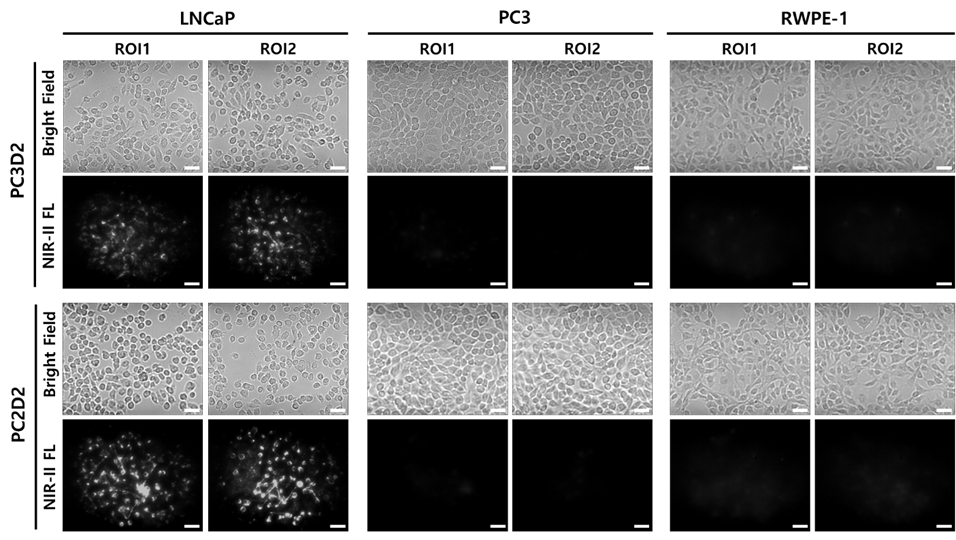


**Figure S14.** **Selective NIR-II Fluorescence of PEG-Functionalized ssDNA-SWCNT Probes in LNCaP Cells.** Bright-field and NIR-II fluorescence images of LNCaP, PC3, and normal prostate epithelial RWPE-1 cells incubated with PEG-functionalized ssDNA-SWCNT probes in a 2D monolayer culture (scale bar = 20 μm). Minimal fluorescence signal was detected across all PC3 and RWPE-1, indicating no appreciable probe binding or uptake in another prostate cancer cells and normal epithelial cells, in contrast to the strong signals observed in LNCaP.


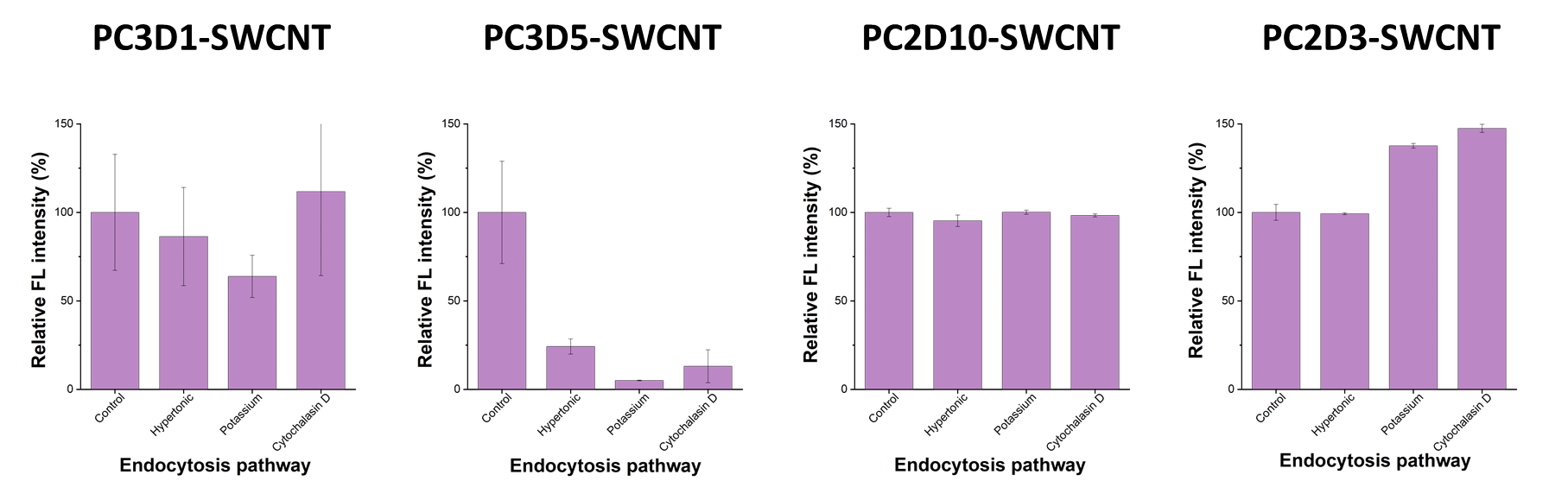


**Figure S15. Quantitative analysis of cellular uptake for probes.** PC2D10- and PC2D3-SWCNT showed fluorescence similar to the control, suggesting minimal entry or other pathways like receptor-mediated endocytosis. In contrast, PC3D1-SWCNT was internalized through clathrin-mediated endocytosis, and PC3D5-SWCNT has dominated uptake via clathrin-mediated pathways, phagocytosis, and macropinocytosis.


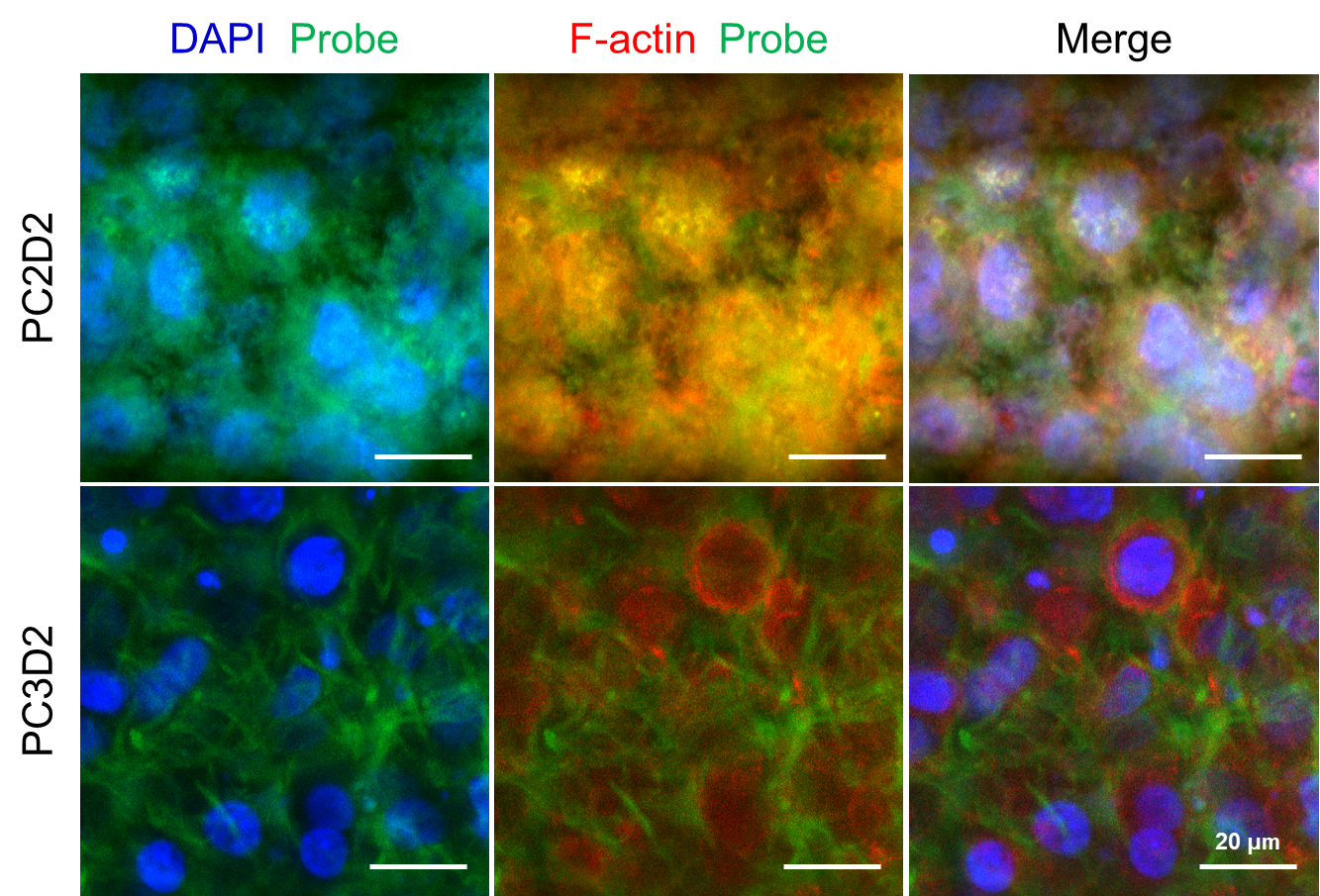


**Figure S16. Differential Intracellular Localization of DNA/SWCNT Probes in LNCaP Cells.** Confocal fluorescence images showing differential intracellular localization of DNA/SWCNT probes in LNCaP cells. LNCaP cells were treated with either PC2D2/SWCNT (bottom row) or PC3D2/SWCNT (top row) and stained with DAPI (nucleus, blue) and F-actin (cytoskeleton, red). The DNA/SWCNT probe fluorescence is shown in green. Confocal Z-stack imaging reveals that PC2D2/SWCNT exhibits strong co-localization with both the nucleus and cytoskeleton, suggesting efficient cellular uptake. In contrast, PC3D2/SWCNT shows limited overlap with intracellular structures, and the fluorescence signal appears primarily at or near the cell surface. (Scale bars = 20 μm)


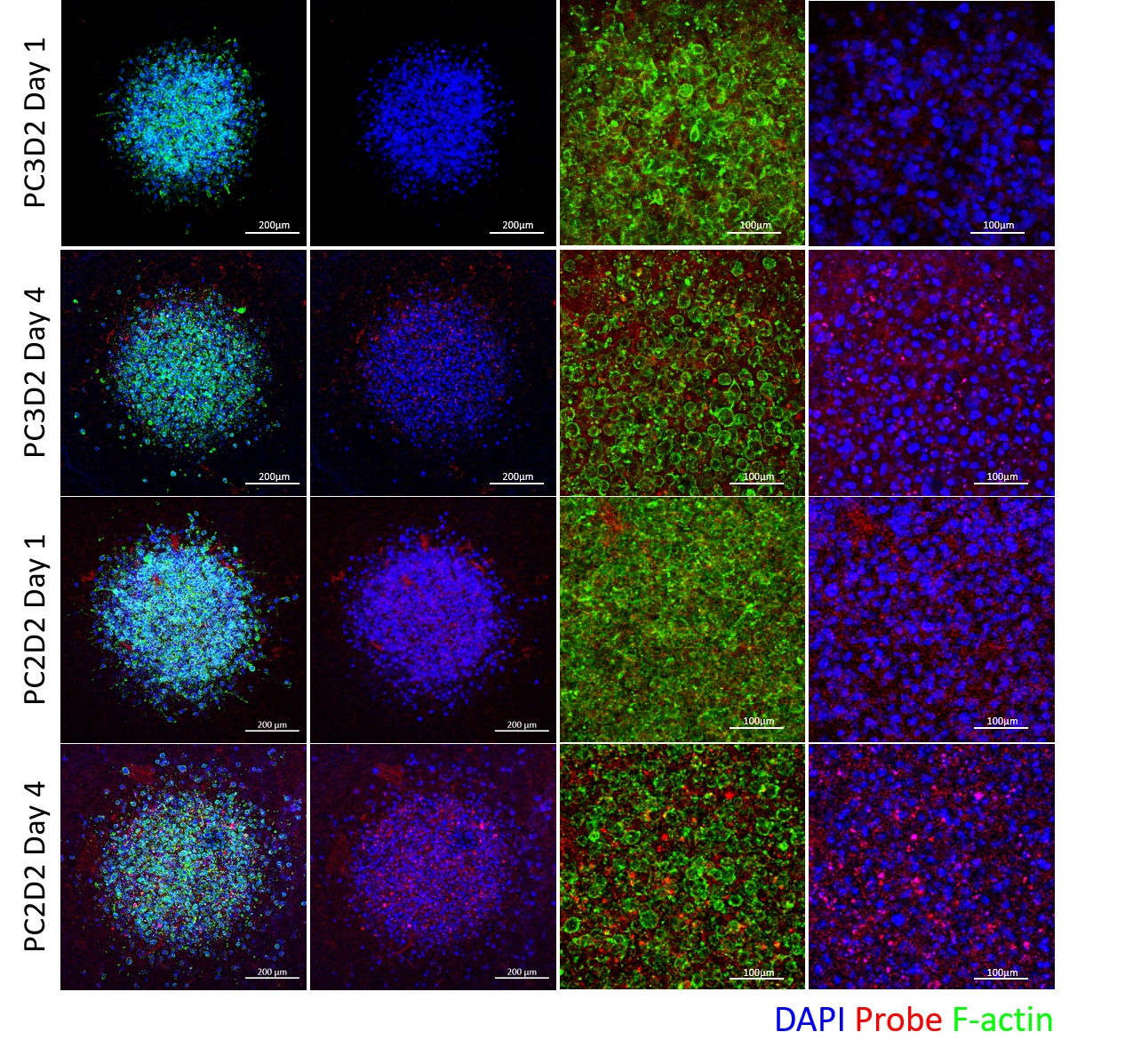


**Figure S17.** Confocal imaging for the probe binding verification of PC3D2 and PC2D2 probes over time. Confocal microscopy images showing the binding of PC3D2 and PC2D2 probes on Days 1 and 4. The cells were stained with DAPI (blue), the probe (red), and F-actin (green) to visualize nuclear localization, probe binding, and cytoskeletal structure. The differences in probe binding between PC3D2 and PC2D2 were observed over time. Scale bars: 200 μm (left), 100 μm (right).


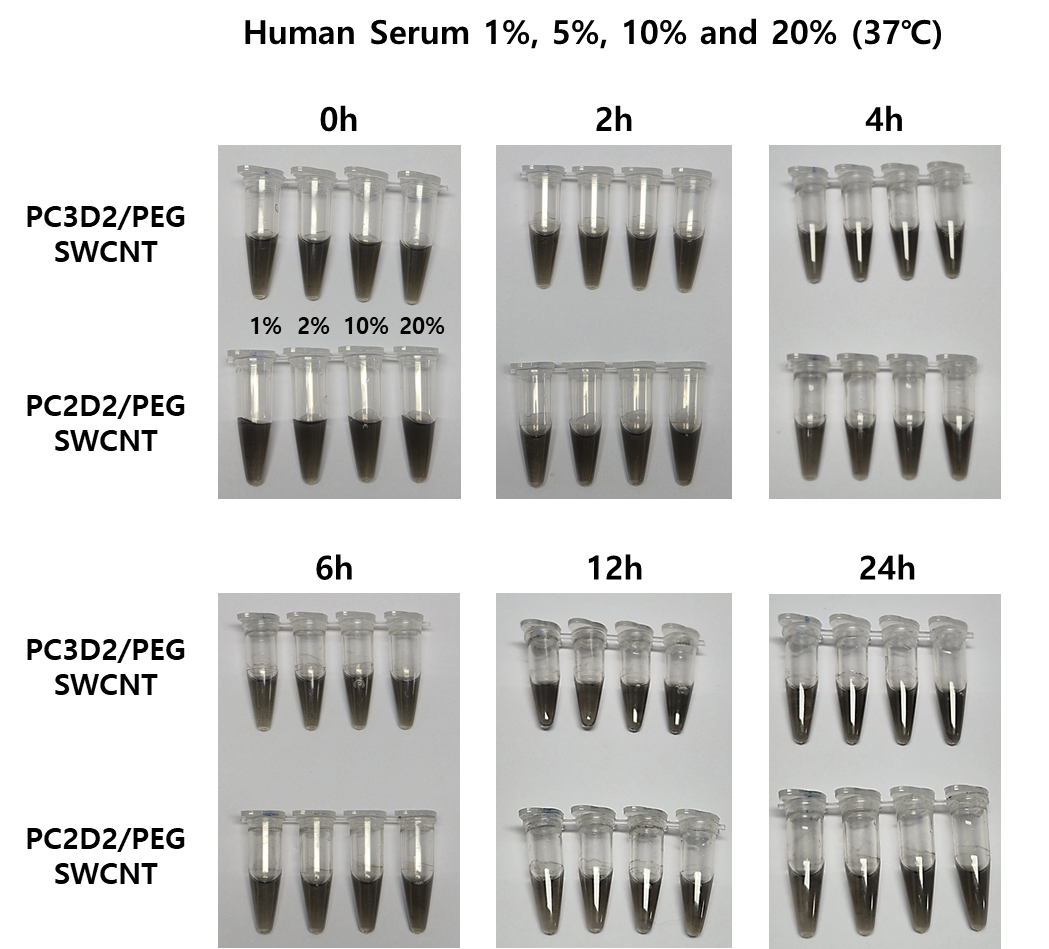


**Figure S18. Stability of probes in human serum.** The images showing the dispersive stability of PC3D2 and PC2D2/PL-PEG-SWCNT constructs incubated in 1%, 5%, 10%, and 20% human serum at 37 °C over time (0, 2, 4, 6, 12 and 24 h). Across all serum concentrations and time points, the probes maintained overall dispersive stability, with only minor wall-associated accumulation observed after 6 h and no detectable bulk aggregation or precipitation.


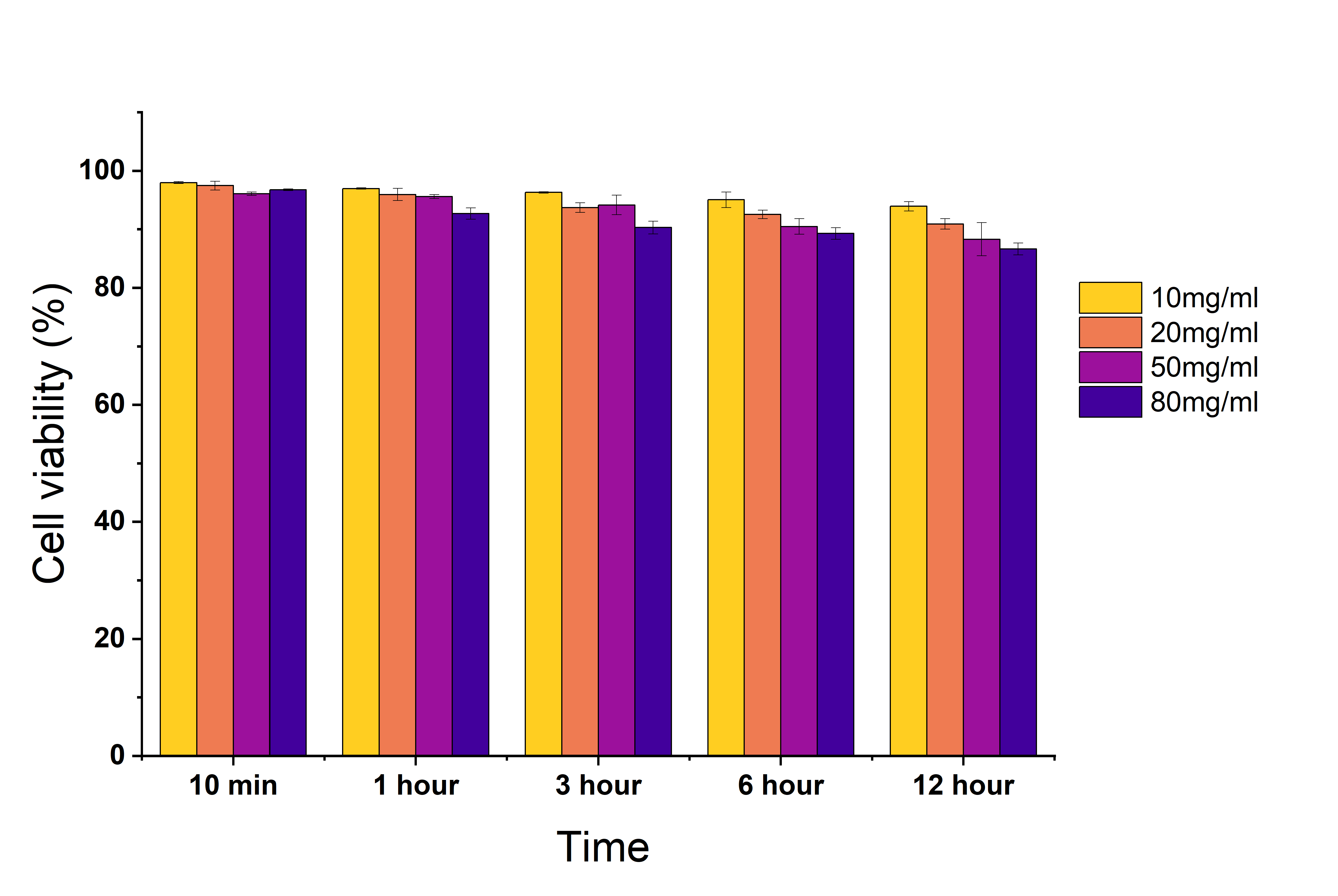


**Figure S19. Cell viability of LNCaP cells.** LNCaP cells were treated with varying concentrations of PEG/ssDNA-SWCNT probes (10, 20, 50, and 80 mg/L) over different time points (10 min, 1 h, 3 h, 6 h, and 12 h). Although the cell viability decreased slightly at higher concentrations and longer exposure times, it remained above 90%, indicating minimal cytotoxicity of the probes.


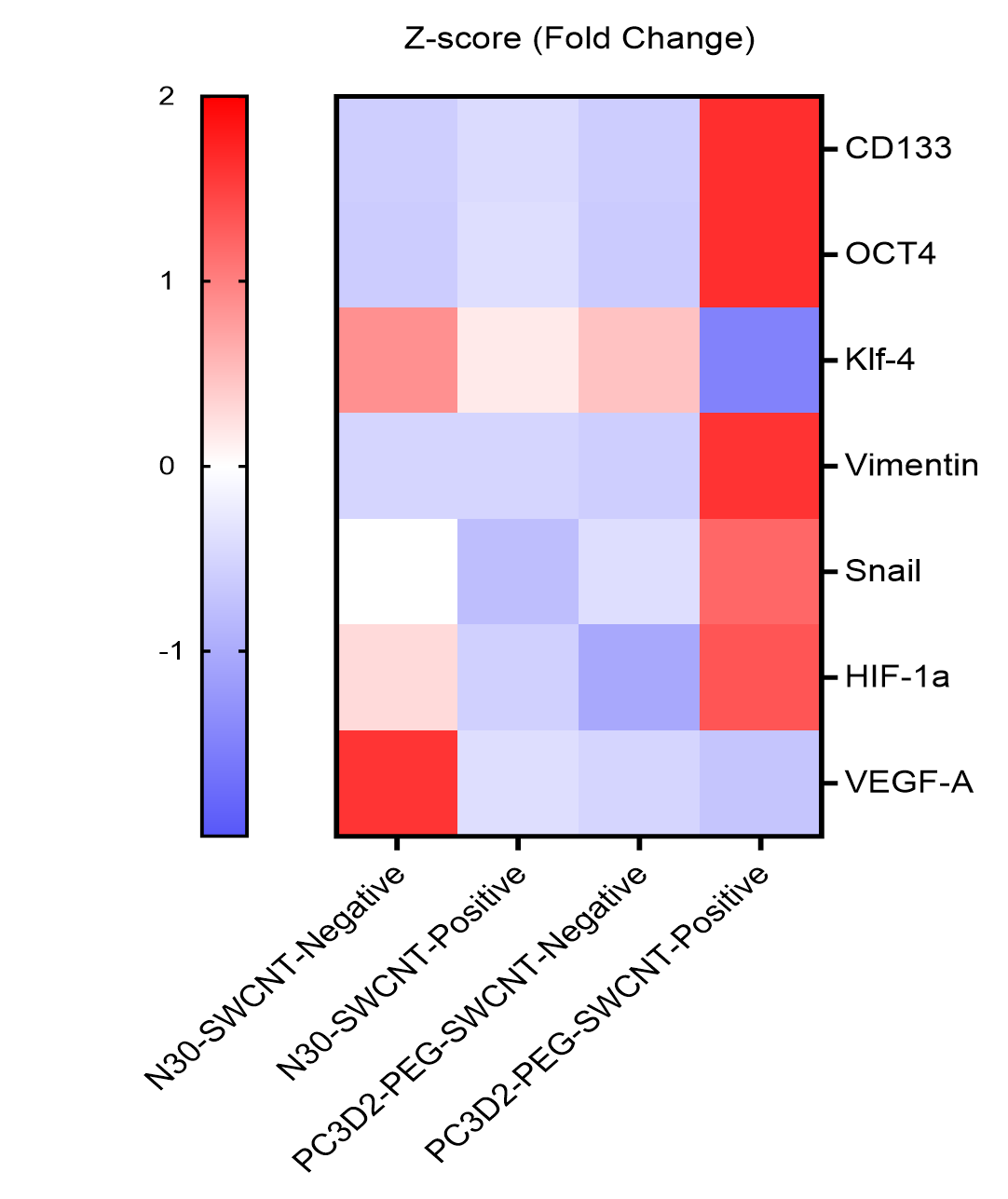


**Figure S20. Gene expression profiles of N30 and PC3D2 probe-positive and negative populations within LNCaP spheroids. (a).** Differential expression of stemness, EMT, and hypoxia markers in probe-positive and negative populations


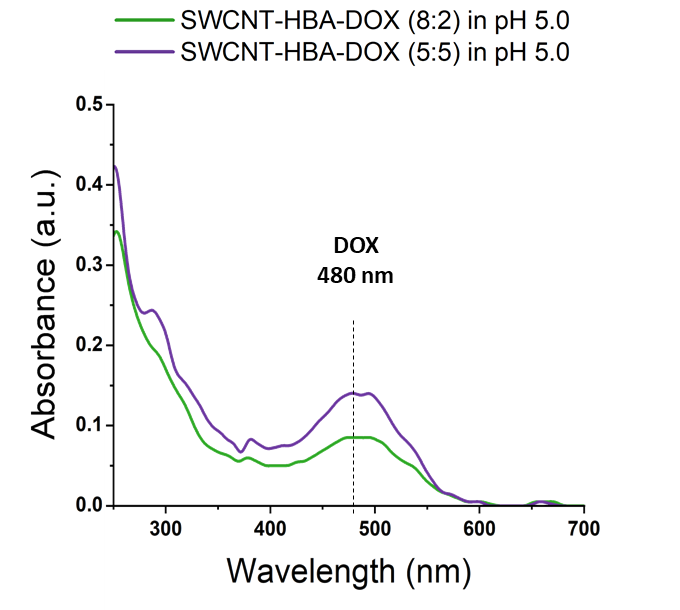


**Figure S21. UV-vis absorbance spectra of WCNT-HBA-DOX conjugates under acidic conditions (pH 5.0).** UV-Vis spectra were acquired to confirm the presence of doxorubicin (DOX) in SWCNT-HBA-DOX conjugates with varying ssDNA:NH_2_-(ACG)_6_ ratios (8:2 in green, 5:5 in purple). The characteristic absorbance peak of DOX at 480 nm in pH 5.0, mimicking the acidic tumor microenvironment. The spectral intensity differences reflect the relative loading and release potential of DOX under these conditions.


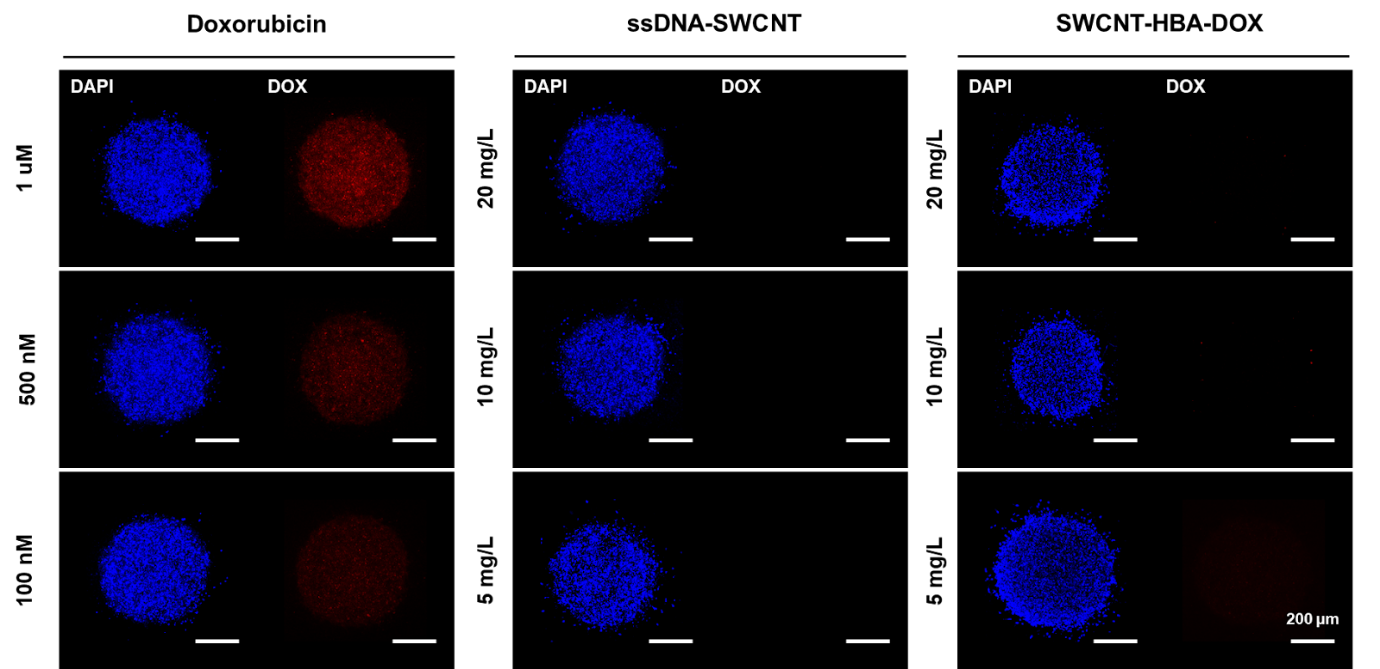


**Figure S22.** **Doxorubicin accumulation in RWPE-1 3D spheroids**. Evaluation of doxorubicin accumulation in RWPE1 3D spheroids treated with PC2D2/SWCNT-HBA-DOX and controls. Fluorescence microscopy images showing doxorubicin accumulation after 24-hour treatment with DAPI (blue) and doxorubicin (DOX, red) fluorescence. he leftmost column represents spheroids treated with free DOX, showing strong red fluorescence due to nonspecific accumulation. The second and third columns show spheroids treated with drug-loaded PC2D2/SWCNT-HBA-DOX and unloaded PC2D2/SWCNT probes, respectively. Both groups exhibited minimal DOX fluorescence, suggesting low nonspecific uptake in non-cancerous spheroids (scale bar = 200 μm).


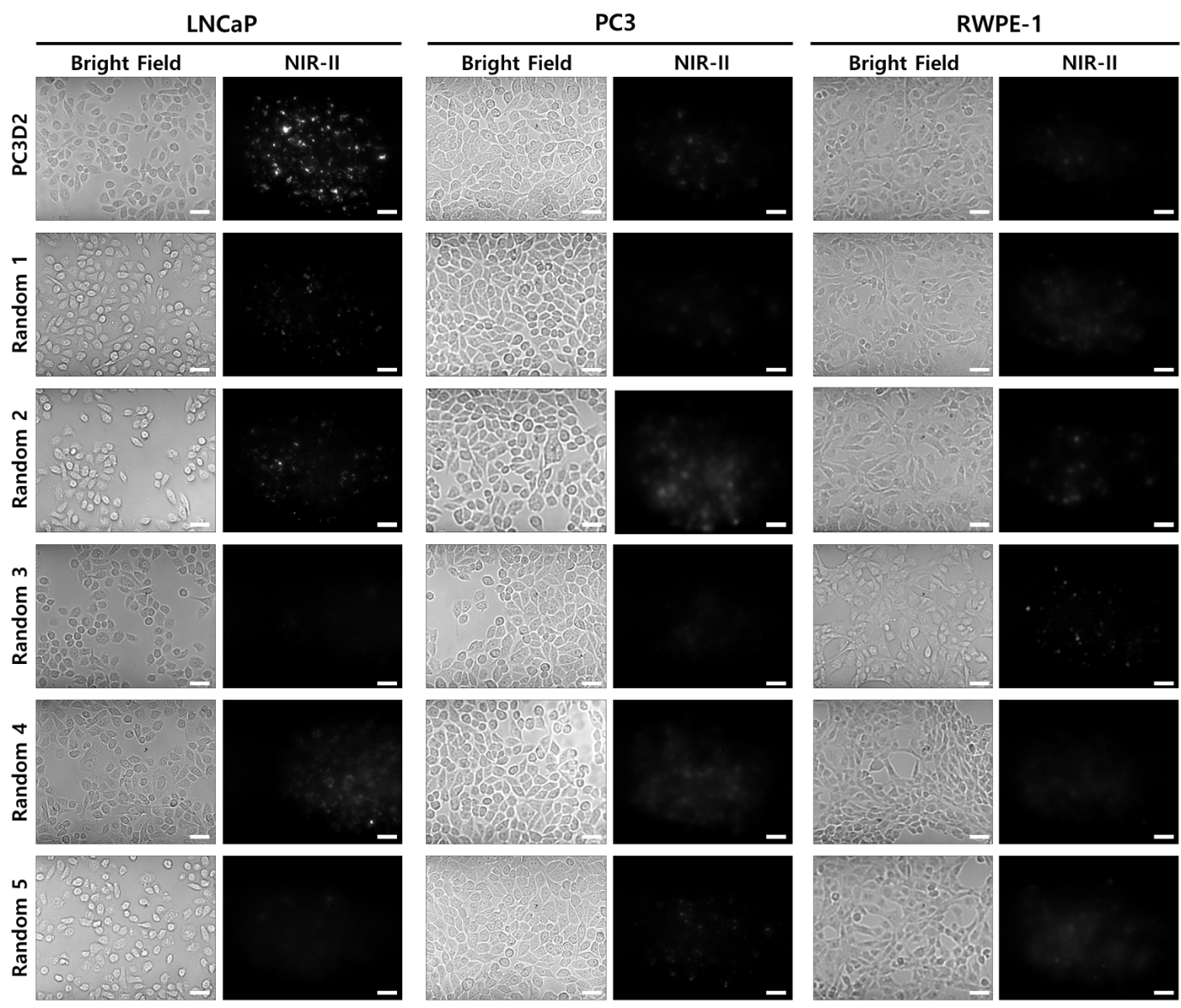

**Figure S23. Comparison of NIR-ll FL signals of randomly selected DNA-wrapped SWCNT probes with LNCap, PC3 and RWPE-1 cells.** Bright field (left of each pair) and corresponding NIR-II FL images (right of each pair) of LNCaP cells incubated with DNA/SWCNT hybrids using five randomly selected ssDNA sequences (not included in the screening library), compared with PC3D2/SWCNT probes. All samples were treated at a concentration of 20 mg/L DNA/SWCNT. Strong fluorescence was observed only for PC3D2, whereas the five random sequences showed negligible NIR-II signal, close to background levels. These results support the necessity of high-throughput screening to identify sequences with high targeting capability (scale bar = 20 μm).

| **ID** | **DNA Sequence** |
| --- | --- |
| Random 1 | GATGCCCTATTGTCGGAGATAGCCACCTGG |
| Random 2 | ACCGTGACTTACCGAATCCGTTTGCTGGAC |
| Random 3 | AAACCTTGAATCTGCTTTAACGAACCTATG |
| Random 4 | CACTCCCCTGGAAAGAGGGAGAAGGGGCAG |
| Random 5 | CGGCGTTAATGTGTAGAGCTTTGTGTGCTA |


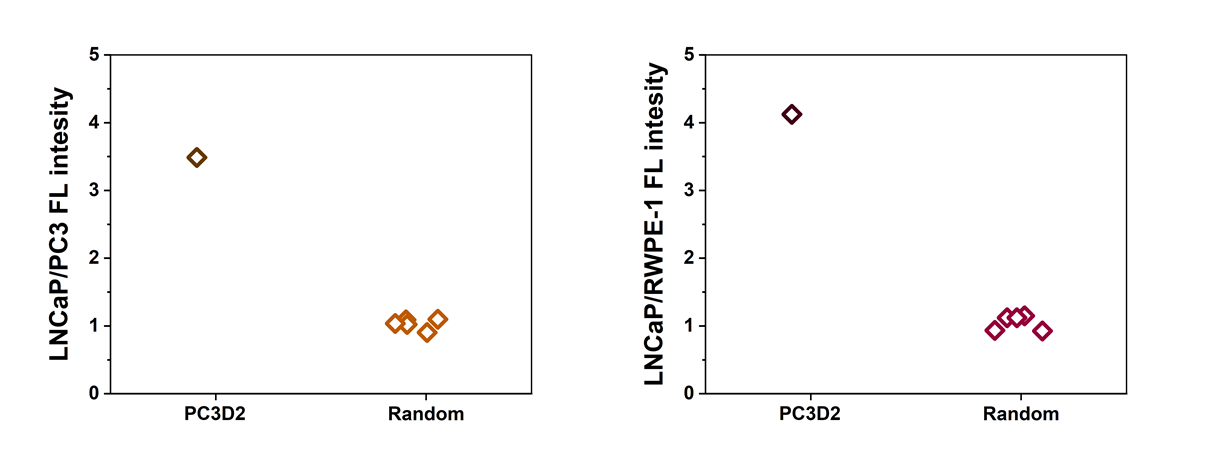


**Figure S24. Cellular Selectivity of PC3D2 versus Random DNA Sequences.** Comparative analysis of cellular selectivity between the PC3D2 DNA sequence and five random DNA sequences for LNCaP cells. Fluorescence intensity ratios of LNCaP relative to PC3 (left) and RWPE-1 (right) cells are shown for PC3D2/SWCNT probes and random-sequence/SWCNT controls. The PC3D2 sequence exhibits a markedly higher fluorescence ratio in LNCaP cells compared to both PC3 and RWPE-1 cells, indicating enhanced selectivity toward LNCaP cells.
